# Care pathways at end-of-life for cancer decedents: registry based analyses of the living situation, healthcare utilization and costs for all cancer decedents in Norway in 2009-2013 during their last 6 months of life

**DOI:** 10.1186/s12913-022-08526-w

**Published:** 2022-10-01

**Authors:** Gudrun Bjørnelv, Terje P. Hagen, Leena Forma, Eline Aas

**Affiliations:** 1 Department of Health Management and Health Economics, Institute of Health and Society, University of Oslo, Oslo, Norway; 2 Department of Public Health and Nursing, Norwegian University of Science and Technology, Trondheim, Norway; 3 Faculty of Social Sciences, Tampere University, Tampere, Finland; 4 Laurea University of Applied Sciences, Vantaa, Finland; 5 Division for Health Services, Institute of Public Health, Oslo, Norway

**Keywords:** Cost and cost analysis, Neoplasms, End-of-life

## Abstract

**Background:**

Research on end-of-life care is often fragmented, focusing on one level of healthcare or on a particular patient subgroup. Our aim was to describe the complete care pathways of all cancer decedents in Norway during the last six months of life.

**Methods:**

We used six national registries linked at patient level and including all cancer decedents in Norway between 2009-2013 to describe patient use of secondary, primary-, and home- and community-based care. We described patient’s car pathway, including patients living situation, healthcare utilization, and costs. We then estimated how cancer type, individual and sociodemographic characteristics, and access to informal care influenced the care pathways. Regression models were used depending on the outcome, i.e., negative binomial (for healthcare utilization) and generalized linear models (for healthcare costs).

**Results:**

In total, 52,926 patients were included who died of lung (16%), colorectal (12%), prostate (9%), breast (6%), cervical (1%) or other (56%) cancers. On average, patients spent 123 days at home, 24 days in hospital, 16 days in short-term care and 24 days in long-term care during their last 6 months of life. Healthcare utilization increased towards end-of-life. Total costs were high (on average, NOK 379,801). 60% of the total costs were in the secondary care setting, 3% in the primary care setting, and 37% in the home- and community-based care setting. Age (total cost-range NOK 361,363-418,618) and marital status (total cost-range NOK354,100-411,047) were stronger determining factors of care pathway than cancer type (total cost-range NOK341,318- 392,655). When patients died of cancer types requiring higher amounts of secondary care (e.g., cervical cancer), there was a corresponding lower utilization of primary, and home- and community-based care, and vice versa.

**Conclusion:**

Cancer patient’s care pathways at end-of-life are more strongly associated with age and access to informal care than underlying type of cancer. More care in one care setting (e.g., the secondary care) is associated with less care in other settings (primary- and home- and community based care setting) as demonstrated by the substitution between the different levels of care in this study. Care at end-of-life should therefore not be evaluated in one healthcare level alone since this might bias results and lead to suboptimal priorities.

**Supplementary Information:**

The online version contains supplementary material available at 10.1186/s12913-022-08526-w.

## Background

Relative to the healthcare experiences of cancer patients immediately after diagnosis, little is known about where and how cancer patients receive treatment at the end of their lives, when no curative treatments are available [1, 2]. Nonetheless, there is a substantial need for healthcare services during this phase of illness as well. [3–5]. To both predict the expected future care burden, and to improve end-of-life care, we need more information about where cancer patients receive care during their last months of life and what factors drive those outcomes.

The existing papers on healthcare costs and healthcare utilization incurred by cancer patients at the end-of-life demonstrate a variety of limitations, including single aspects of care (e.g., only hospital costs) [6–9], small samples [6, 10, 11], selected cohorts (e.g., only the elderly) [12, 13] or focusing on specific cancers [14–19].

Increasingly, the focus on quality end-of-life care has emphasized integration as a key element. Patients prefer to be cared for, and to die at home [20, 21], which requires close collaboration between secondary-, primary-, and home- and community-based care providers [20–25]. Without evaluating all levels of care simultaneously it is impossible to have a clear overview of the patient and family experience. For example, if only the quantity of in-hospital care is evaluated, some patients may appear to need little end-of-life care, while in reality they might have received substantial amounts of home based services and/or informal care [13].

Existing literature is also limited in its lack of recognition of the heterogeneity of patient experience based on type of cancer, comorbidities, age, gender, sociodemographic characteristics, and access to informal care. Lung-, colon-, rectal-, prostate-, and breast cancer are the most common causes of cancer death [26]. Cancer type determines treatment regimen and attendant side effects. It also influences the most common sites of metastasis, which influence symptom burden. For example, the presence of metastatic lesions in bone predicts a greater likelihood of pain. Consequently, cancer type may impact care needs in the last six months of life. Experiences might also differ by cancer type if the populations in which the cancers occur are different. For example, cervical cancer occurs in younger women, and prostate cancer in older men. As age increases, so do the likelihood of frailty and comorbidities, increasing the need for healthcare regardless of cancer type. There are also sociodemographic characteristics that influence the prevalence of some cancers. For example, lung cancer is more prevalent in persons with low education, while breast cancer is more prevalent in persons with high education [27, 28]. Education is known to be an independent factor for increasing healthcare utilization – those with more education use more healthcare services than others [29]. Finally, factors such as age, gender, and marital status, which may differ among cancer patients, all impact the availability of informal care. Access to informal caregivers is a key factor in the ability of patients to remain in the home, and informal caregivers also serve as patient advocates [30–34].

Cancer patients’ total need for, and use of, care at all levels of the healthcare sector during the last six months of their lives probably depends on many interacting factors in ways that have not been fully characterized. For example, a widowed prostate cancer patient with a high age and many comorbidities might need a high level of formal care and may for instance need to reside in a nursing home. A younger, married cervical cancer patient with no comorbidities might be able to stay at home, and thus, require more home-based and informal care.

Our aim in the current paper is to gain deeper knowledge about the living situation, healthcare utilization and costs of cancer patients at end-of-life. We will do this by first describing the complete care pathways of all cancer decedents during their last six months of life. Next, we will examine how the care pathways are influenced by cancer type, individual- and sociodemographic characteristics, and access to informal care. We will use national registries covering all Norwegians who died of cancer between 2009–2013. For the purposes of this study, end-of-life was defined as the last six months of life, and care pathways included information on the living situation and healthcare utilization at the secondary, primary-, and home- and community-based care level. We described care for patients depending on their underlying cause of death, grouped as lung-, colorectal-, prostate-, breast-, cervix-, or other cancers.

## Methods

### Patient population

We identified all individuals who died in Norway between 2009 and 2013 from the Norwegian Causes of Death Registry (CDR), which covers 100% of the Norwegian population [35]. From the registry, we gained individual-level information about the cause of death as noted by the physician who completed their death certificate. All individuals were linked to the Cancer Registry of Norway (CRN), from which we obtained information about the type (ICD-7 codes) and year (between 1951 and 2013) of their primary cancer diagnosis [36]. We grouped patients into those who died from the most common cancers in Norway: lung cancer, colorectal cancer, prostate cancer, and breast cancer. We also included those who died from cervical cancer as a separate group.^1^ All other cancer types were grouped into the category other cancer deaths. This classification was done by using information from both registries. Individual were only classified into a specific group if the cause of death reported in the CDR coincided with the primary cancer diagnosis reported in the CNR.

### Healthcare utilization – secondary, primary-, and home- and community-based care

The Norwegian Patient Registry (NPR) includes all hospital claims data for Norway [37]. Each time a treatment is provided to a patient, a claim is sent to the NPR. The claims include diagnosis, treatment and procedures and is broken down by diagnosis-related group (DRG), of which there are 900. The DRG assigned determines the amount of money a hospital will be reimbursed by for the patient’s visit. It is estimated based on reports from several hospitals who perform the procedure and is intended to cover both direct and indirect costs to the hospital (including complications and overhead), but excludes the costs of laboratory studies, radiology, and any co-payments. The DRG cost is assumed to reflect the mean cost of a treatment [38]. We derived information from NPR on all treatments cancer decedents had received in hospitals (grouped as inpatient treatments or outpatient treatments), the total number of days patients spent in the hospital, and the total costs of hospital treatments (based on the DRG costs), during their last six months of life.

The Control and Payment of Health Reimbursement registry (KUHR) includes information on all treatment people in Norway receive from primary care providers [39]. Each time a patient receives treatment, a claim is sent to the Norwegian Health Economics Administration (HELFO) and then stored in the KUHR database. Claims include diagnosis and the treatment as well as co-pay information. Treatments are coded according to current tariffs [40]. Each code in the tariffs has a cost attached, indicating the reimbursement that the care provider receives for the treatment from HELFO. The reimbursement is intended to cover the cost of the treatment, excluding basic costs that are provided through block grants, and patient co-payments. From KUHR, we received information on all claims sent from general practitioners (GPs) and local emergency rooms (ERs),^2^ including information about the amounts reimbursed and out-of-pocket payments. We also received information from KUHR on claims related to laboratory and radiology services provided at hospitals, and all patient co-payments paid to hospitals. The latter (laboratory, radiology, and co-payments) was used to estimate the total cost of hospital treatment (see section on healthcare costs, below).

Home- and community-based services are funded from the municipalities’ global budgets and hence, no individual claims data are available. However, for the purpose of research, quality assurance, future planning and control, all municipalities are required to gather information on the number of patients who have applied for and/or received home and community-based care. The information is gathered in the Individual-Based Nursing and Care Statistics Registry called IPLOS [41]. From IPLOS, we obtained information on how many days the cancer decedents lived in institutions (distinguishing between short-term and long-term institutions). We also obtained information on whether patients received home-based care, either practical assistance or nursing assistance, and the magnitude of that care measured as the total number of hours of care patients received.

To estimate the number of days each patient spent at home, we subtracted the number of days the patient spent in hospital, in long-term institutions, and in short-term institutions, from the total number of days during 6 months (i.e., 180 days). Since a patient’s place in a long-term institution is not used by others if she or he is absent (due to for example hospitalization), we allowed for an overlap between hospital stays and long-term institutional stays.

### Healthcare costs

We estimated the costs of secondary and primary care using information from DRG-codes and claims from NPR and KUHR. The costs of secondary care were estimated as 100% of the DRG cost. We estimated the total costs of care from GP consultations, local ER visits, and radiology and laboratory services (in KUHR) by summarizing the total reimbursement and patient co-payments, and dividing this by 0.3—since the Norwegian Directorate of Health estimated that the reimbursement claims and patient co-payments in KUHR summarized to approximately 30% of the total cost of treatments [42–44]. We estimated the costs of living in an institution by multiplying the number of days a patient stayed in a short- or long-term institution, by the corrected gross operating expenses published by Statistics Norway (SSB) as a part of the Municipality-State-Reporting (KOSTRA) [45]. To estimate the cost of practical assistance and nursing assistance, we multiplied the number of hours that patients received with practical assistance or nursing care, by the mean cost of care per hour as estimated by Langeland et al. [46]. Costs were estimated in 2013 Norwegian Kroner (NOK). NOK 1 was approximately USD 0.17 and EURO 0.13.

### Individual characteristics

SSB provided information on the populations’ age at death, sex, highest level of education, income, and marital status. Age was grouped as below 50 years, 50–69, 60–69, 70–79, 80–89, or > 90; education as primary school (0–10 years), secondary school (11–13 years), or higher education (> 14 years), and income in quartiles, by gender, for the entire cohort of patients dying (all dying in Norway) between 2008 and 2013. From NPR, we received information on the comorbidities of patients six months prior to death, estimated and classified into the Charlson Comorbidity Index (CCI) based on hospital records (ICD-10 codes), including both the primary and secondary diagnoses, from 18 to 7 months before death [47, 48]. Comorbidities were grouped into mild/moderate (0–4) or severe (> 5) comorbidities. Individuals that did not have any hospital contacts 18 to 7 months prior to their death, were assumed to have mild/moderate comorbidities. Marital status six months prior to death was divided into the three groups never married, those currently married/registered as partner, and those previously married, meaning, divorcees, widow/widowers or previously registered as partner. We used marital status as a proxy for access to informal care.

### Statistics

We used descriptive statistics to display individual, sociodemographic and disease characteristics for the total population, and for the population according to their cause of death. Differences between groups (type of cancer decedent) were tested using chi-square statistics.

For all levels of healthcare utilization, we assumed that a missing registration meant no utilization and set the missing values as zero. Because of data anonymization requirements, information on healthcare utilization was provided to us for three periods: 6–4 months prior to death, 3–2 months prior to death, and 1 month prior to death. When describing care as patients approach death, we defined resource use as the mean use per month, per period, e.g., use during the 6 – 4-month period was divided by 3.

To estimate the effect that type of cancer, individual- and sociodemographic characteristics, and access to informal care had on living situation, healthcare utilization, and costs, we ran separate multivariate regression models for all outcome variables. In these analyses, we used living situation (i.e., living in hospital, in short or long-term nursing home, or at home), healthcare utilization and costs during the entire 6-month period prior to death as outcome variables. In the analyses, we included the variables: type of cancer (prostate cancer as reference category), age (below 60 as reference category), sex (male as reference category), marital status (never married as reference category), Charlson comorbidity index (mild/moderate as reference category), education (primary school as reference category) and income (lowest quartile as reference category). We also adjusted for years since diagnosis and year of death (distinguishing between those dying in 2009–2010 and 2011–2013).

Regression model selection was performed by first assessing the characteristics of the outcome variables, and then, for each outcome variable, choosing the best model from a set of appropriate models. Models were selected using the Akaike Information Criterion (AIC) and the Bayesian Information Criterion (BIC) [49]. For variables on healthcare utilization and living situation, that were non-negatively skewed integer numbers (count variables), we chose between: Poisson, the negative binomial 1, and the negative binomial 2 models. For two of the outcome variables for living situation (days in a long-term institution and days at home) neither the Poisson nor negative binomial model was appropriate because of the large number of zeroes in the variables, with a heavy tail of increasing counts at higher values. For these two outcomes, we therefore tested two-part models using logistic regression in the first part and ordinary least square regressions in the second part. For variables on costs, that were non-negatively skewed continuous numbers, we ran generalized linear models (GLM). For all models, we tested different links (identity, log, and power) and distributional families (Gaussian, inverse Gaussian, and Gamma). We used robust standard errors.

We present results from the regression models as the average marginal effects (AME), displaying variables on their original scale [50].

## Results

### Patients

Between 2009 and 2013, 52,926 people died from cancer in Norway – of these – 16% died from lung cancer, 12% from colorectal cancer, 9% from prostate cancer, 6% from breast cancer and 1% from cervical cancer. The rest (56%) died from other cancer types. Among those who died from other cancers, the five most common primary diagnoses were: 1) cancers in the hematopoietic system (*n* = 2,563, 9%), or, malignant neoplasms of 2) the bladder and other urinary organs (2,014, 7%), 3) the pancreas (1,432, 5%), 4) the ovary, fallopian tube or broad ligament (*N* = 1,431, 5%), or 5) brain or other parts of the nervous system (*n* = 1,348, 5%).

The average age at death was 80–84 years. The majority (44%) had completed secondary school while 14% had completed higher education. People were most frequently married or with a partner (49%). Most decedents had been diagnosed less than a year before they died (34%) (Table 1).

**Table 1:** Descriptive statistics of the entire cohort, and when divided between patients cause of death

	**All (*n* = 52,926)**		**Lung (*n* = 8,701)**		**Colorectal (*n* = 6,468)**		**Prostate (*n* = 4,658)**		**Breast (*n* = 3,123)**		**Cervix (*n* = 353)**		**Other (*n* = 29,623)**		
	n	%	n	%	n	%	n	%	n	%	n	%	n	%	p
**Age**															
< *50*	2,351	(0.04)	235	(0.03)	212	(0.03)	3	(0.00)	293	(0.09)	86	(0.24)	1,522	(0.05)	< .001
*50–59*	4,917	(0.09)	1,041	(0.12)	527	(0.08)	69	(0.01)	483	(0.15)	61	(0.17)	2,736	(0.09)	
*60–64*	5,073	(0.10)	1,241	(0.14)	565	(0.09)	171	(0.04)	322	(0.10)	34	(0.10)	2,740	(0.09)	
*65–69*	6,626	(0.13)	1,498	(0.17)	796	(0.12)	352	(0.08)	349	(0.11)	33	(0.09)	3,598	(0.12)	
*70–74*	6,584	(0.12)	1,365	(0.16)	788	(0.12)	482	(0.10)	299	(0.10)	24	(0.07)	3,626	(0.12)	
*75–79*	7,578	(0.14)	1,321	(0.15)	933	(0.14)	760	(0.16)	304	(0.10)	35	(0.10)	4,225	(0.14)	
*80–84*	8,702	(0.16)	1,189	(0.14)	1,080	(0.17)	1,138	(0.24)	344	(0.11)	35	(0.10)	4,916	(0.17)	
*85–89*	6,944	(0.13)	611	(0.07)	909	(0.14)	1,054	(0.23)	364	(0.12)	30	(0.08)	3,976	(0.13)	
> *90*	4,151	(0.08)	200	(0.02)	658	(0.10)	629	(0.14)	365	(0.12)	15	(0.04)	2,284	(0.08)	
**Gender** (females) ^*^	24,538	(0.46)	3,693	(0.42)	3,227	(0.50)	0	(0.00)	3,100	(0.99)	353	(1.00)	14,165	(0.48)	< .001
**Education**															
*Primary school*	20,925	(0.41)	4,028	(0.48)	2,572	(0.41)	1,743	(0.37)	1,094	(0.39)	153	(0.57)	11,335	(0.40)	< .001
*Secondary school*	22,409	(0.44)	3,636	(0.43)	2,785	(0.45)	2,046	(0.44)	1,271	(0.45)	82	(0.31)	12,589	(0.45)	
*Higher education*	7,241	(0.14)	802	(0.09)	899	(0.14)	866	(0.19)	465	(0.16)	32	(0.12)	4,177	(0.15)	
**Marital status**															
*Never married*	5,172	(0.10)	812	(0.09)	636	(0.10)	343	(0.07)	318	(0.10)	59	(0.17)	3,004	(0.10)	< .001
*Married/ partner*	26,090	(0.49)	4,358	(0.50)	3,027	(0.47)	2,790	(0.60)	1,289	(0.41)	122	(0.35)	14,504	(0.49)	
*Previously married/ pratner*	21,594	(0.41)	3,516	(0.40)	2,799	(0.43)	1,521	(0.33)	1,509	(0.48)	171	(0.49)	12,078	(0.41)	
CCI ^**^ (mild/ moderate)	39,138	(0.74)	7,119	(0.82)	4,060	(0.63)	2,980	(0.64)	1,567	(0.50)	191	(0.54)	23,221	(0.78)	< .001
**Years since diagnosis**															
*0*	17,970	(0.34)	4,930	(0.57)	2,269	(0.35)	468	(0.10)	309	(0.10)	81	(0.23)	9,913	(0.33)	< .001
*1*	9,614	(0.18)	2,306	(0.27)	1,372	(0.21)	471	(0.10)	305	(0.10)	75	(0.21)	5,085	(0.17)	
*2*	4,827	(0.09)	717	(0.08)	813	(0.13)	454	(0.10)	271	(0.09)	36	(0.10)	2,536	(0.09)	
*3*	3,228	(0.06)	268	(0.03)	534	(0.08)	434	(0.09)	236	(0.08)	36	(0.10)	1,720	(0.06)	
*4*	2,507	(0.05)	145	(0.02)	366	(0.06)	378	(0.08)	236	(0.08)	23	(0.07)	1,359	(0.05)	
*5*	1,847	(0.03)	75	(0.01)	227	(0.04)	338	(0.07)	216	(0.07)	16	(0.05)	975	(0.03)	
*6–10*	5,965	(0.11)	163	(0.02)	536	(0.08)	1,215	(0.26)	690	(0.22)	22	(0.06)	3,339	(0.11)	
*11–20*	4,657	(0.09)	78	(0.01)	255	(0.04)	839	(0.18)	597	(0.19)	27	(0.08)	2,861	(0.10)	
> *21*	2,311	(0.04)	19	(0.00)	96	(0.01)	61	(0.01)	263	(0.08)	37	(0.10)	1,835	(0.06)	
**Year of death**															
*2009*	10,358	(0.20)	1,666	(0.19)	1,274	(0.20)	943	(0.20)	655	(0.21)	84	(0.24)	5,736	(0.19)	0.62
*2010*	10,706	(0.20)	1,761	(0.20)	1,308	(0.20)	936	(0.20)	629	(0.20)	74	(0.21)	5,998	(0.20)	
*2011*	10,622	(0.20)	1,759	(0.20)	1,284	(0.20)	951	(0.20)	584	(0.19)	67	(0.19)	5,977	(0.20)	
*2012*	10,604	(0.20)	1,736	(0.20)	1,309	(0.20)	910	(0.20)	622	(0.20)	57	(0.16)	5,970	(0.20)	
*2013*	10,636	(0.20)	1,779	(0.20)	1,293	(0.20)	918	(0.20)	633	(0.20)	71	(0.20)	5,942	(0.20)	

*Numbers given as number of people (n) and percentage of population (%)*

^*^ Although it is rare, a few men are diagnosed with breast cancer each year. Therefore, 123 men are included in the breast cancer group in the table

^**^ CCI charlson comorbidity index

Patient characteristics differed across the different cancer groups (see Table 1). For example, patients dying from breast and cervical cancer were younger, and women, while patients dying from prostate cancer were older, and male. The level of education was lower among those dying from cervical cancer, and they were more often never married. While the majority died within the first two years after diagnosis (lung (83%), colorectal (56%), cervical (44%) and other cancers (51%)) only 20% of patients dying from prostate and breast cancer died within the first two years. These patients more often died 6–10 years (26% prostate and 22% breast) or 11–20 years (18% prostate and 19% breast) after their primary diagnosis. For details, see Table 1.

### Descriptive statistics

#### Living situation

**Fig. 1:**
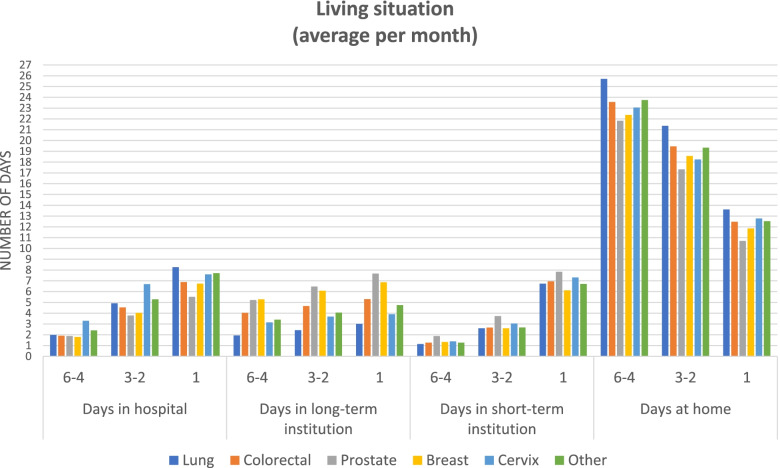
Descriptive statistics of the living situation of patients, reported as the average number of days per month in the 6–4 month, 3–2 month and 1 month period prior to death

On average, patients spent 123 days at home, 24 days in hospital, 16 days in short-term care and 24 days in long-term care during their last 6 months of life. As death approached, patients spent more days in hospital and in short-term institutions. The number of days patients spent in long-term institution was stable, while the number of days patients spent at home decreased. There was minor variation in the living situation across the cancer types (Fig. 1). For details, see Additional file 1, Table S1.

#### Healthcare utilization

**Fig. 2:**
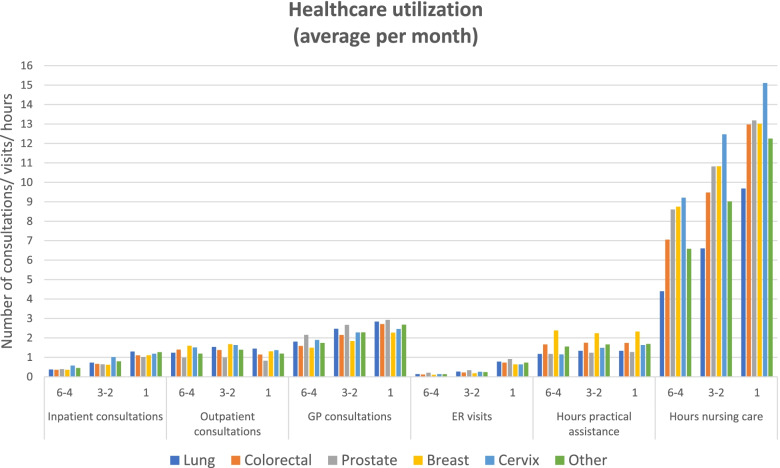
Descriptive statistics of the healthcare utilization of patients, reported as the average number of treatments patients receive per month in the 6–4 month, 3–2 month and 1 month period prior to death

During the last 6 months prior to death, patients had an average of 4 inpatient hospital stays, 8 outpatient consultations, 13 GP-consultations, 2 ER-visits, 9 h of practical assistance and 50 h of nursing assistance. Overall, healthcare utilization (inpatient care, GP-consultations, ER-visits, and hours of practical and nursing assistance) increased towards patients’ end-of-life. The number of outpatient consultations decreased towards patients’ end-of-life. There was minor variation in healthcare utilization between patients depending on their type of cancer (Fig. 2). For details, see Additional file 1, Table S1.

#### Healthcare costs

On average, the total healthcare costs during the 6 months prior to death summed to NOK379,801, of which 60%, 3% and 37% were in the secondary, the primary and the home- and community-based care setting, respectively. Cancer decedents that utilized more secondary care (e.g., cervical cancer – at an average of NOK 267,097) used less primary, and home and community-based care (NOK 11,835 and NOK 150,499, respectively). And vice versa; cancer decedents that had lower use of secondary care (e.g., prostate cancer – at an average of NOK 162,853) used more primary, and home and community-based care (NOK 14,515 and NOK 189,250, respectively). This indicates a substitutionary relationship between the levels of care. For details, see Additional File 1, Table S1.

#### Regression analyses

Results from the regression analyses revealed that after controlling for individual- and sociodemographic factors, the living situation, healthcare utilization, and healthcare costs of cancer decedents varied only slightly depending on the individuals cause of death (type of cancer), Table 2. Individuals living situation depended more on the cancer patients age and access to informal care (marital status), than their underlying cause of death. For details, see Additional file 2, Figures S1,S2,S3,S4,S5,S6,S7,S8,S9,S10,S11,S12,S13, and S14.

**Table 2:** Healthcare utilization and living situation depending on type of cancer death

	**Lung (*n* = 8,701)**			**Colorectal (*n* = 6,468)**			**Prostate (*n* = 4,658)**			**Breast (*n* = 3,123)**			**Cervix (*n* = 353)**			**Other (*n* = 29,623)**		
	AME	95% CI		AME	95% CI		AME	95% CI		AME	95% CI		AME	95% CI		AME	95% CI	
***Inpatient consultations***^1^	3.6	(3.5	3.7)	3.5	(3.4	3.6)	3.7	(3.6	3.9)	3.1	(3.0	3.2)	4.1	(3.8	4.5)	4.1	(4.1	4.2)
***Outpatient consultations***^**2**^	7.6	(7.5	7.8)	7.4	(7.2	7.6)	6.6	(6.4	6.8)	7.7	(7.5	8.0)	5.8	(5.2	6.5)	7.3	(7.2	7.4)
***GP consultations***^2^	13.4	(13.2	13.6)	11.7	(11.4	11.9)	14.0	(13.6	14.4)	10.2	(9.9	10.6)	11.8	(10.5	13.0)	12.6	(12.5	12.7)
***ER visits***^1^	3.1	(3.0	3.2)	2.8	(2.7	2.9)	3.4	(3.3	3.5)	2.6	(2.4	2.7)	2.8	(2.4	3.3)	2.9	(2.9	3.0)
***Hours PA***^2^	10.0	(9.2	10.7)	8.4	(7.7	9.0)	9.2	(8.4	10.1)	8.4	(7.6	9.2)	6.4	(4.8	8.1)	8.6	(8.1	9.2)
***Hours nursing care***^2^	50.1	(48.2	51.9)	49.2	(47.2	51.1)	56.2	(53.6	58.8)	43.0	(40.5	45.6)	48.6	(40.2	57.1)	49.3	(48.0	50.5)
***Days in hospital***^1^	21.3	(21.0	21.7)	21.8	(21.4	22.3)	21.9	(21.2	22.5)	19.3	(18.6	20.1)	24.9	(22.4	27.4)	25.0	(24.7	25.2)
***Days in STI***^2^	17.7	(17.1	18.4)	15.5	(14.9	16.1)	19.7	(18.8	20.7)	13.8	(12.9	14.7)	18.5	(15.0	22.0)	16.4	(16.0	16.7)
***Days in LTI***^2^	21.2	(19.8	22.6)	24.9	(23.4	26.4)	33.4	(31.1	35.7)	32.4	(29.7	35.1)	20.8	(14.1	27.6)	22.8	(22.1	23.4)
***Days at home***^3^	127.9	(126.7	129.2)	123.1	(121.8	124.5)	112.6	(110.8	114.4)	119.4	(117.1	121.7)	118.4	(111.7	125.1)	122.3	(121.6	122.9)
***Secondary care costs***^4^	199,685	(196,452	202,917)	233,471	(228,849	238,093)	183,577	(178,763	188,392)	175,578	(169,879	181,277)	222,772	(200,137	245,408)	232,658	(230,448	234,869)
***Primary care costs***^5^	12,716	(12,436	12,995)	11,350	(11,050	11,649)	13,982	(13,543	14,422)	9,737	(9,310	10,164)	11,538	(9,882	13,193)	12,313	(12,161	12,466)
***HAC based care costs***^4^	128,917	(124,160	133,675)	143,728	(138,421	149,035)	191,283	(183,226	199,339)	151,476	(143,953	158,998)	154,483	(130,857	178,109)	145,196	(142,780	147,612)
***Total costs***^6^	341,318	(336,511	346,125)	392,655	(386,895	398,415)	383,099	(376,291	389,907)	360,437	(351,767	369,106)	383,619	(355,453	411,785)	385,705	(382,855	388,554)

Numbers given as average marginal effect (AME) with 95% confidence intervals. Each row represents one regression analysis. Results from regression analyses using fully adjusted models (including covariates age, gender, marital status, comorbidities, years since diagnosis, period, education and income)

^1^ Negative binomial 2

^2^ Negative binomial 1

^3^ Two part model, logistic regression (first part) GLM with identity link and gaussian distribution (second part)

^4^ GLM with log link and gamma distribution

^5^ GLM with log link and identity distribution

^6^ GLM with identity link and gaussian distribution

*PA* Practical assistance, *STI* short-term institution, *LTI* long-term institution, *HAC* home- and community based care

#### Living situation

The difference in living situation between patients, depending on their type of cancer, varied by 6 days in hospital (min breast/ max other cancers), 6 days in short-term institution (min breast/ max prostate), 13 days in long-term institution (min cervical/ max prostate cancer), 15 days at home (min prostate/ max lung cancer). However, age and access to informal care (marital status) were stronger predictors of patients’ living situation. For example, compared to those below the age 60, those who were 90 years or above spent 21 (95% CI, -22—-20) fewer days in hospital, 9 (95% CI, 8 – 10) more days in short time institutions, 55 (95% CI 52 – 58) more days in long-term institutions and 38 (95% CI, -41—-36) fewer days at home. Compared to the never married, those who were married, on average, spent 3 (95% CI, 2—3) more days in hospital, 5 (95% CI, -6—-4) fewer days in short time institution, 32 (95% CI, -35—-29) fewer days in long-term institution and 29 (95% CI, 27 – 31) more days at home during their last 6 months of life. Education and income were also associated with patients living situation, however, the size of the effect was relatively small between those with highest/ lowest education and income (compared to the effect of age and marital status) (see Additional file 2, Figures S1,S2,S3,S4,S5,S6,S7,S8,S9,S10,S11,S12,S13, and S14).

#### Healthcare utilization

The difference in healthcare utilization between patients, depending on their type of cancer, varied slightly, Table 2. As with patients living situation, the patients age and access to informal care (marital status) were strong predictors of healthcare utilization. For example, those in the oldest age group (90 +) had fewer outpatient consultations (-8, 95% CI -8.3—-7.7) than the youngest age group, while those married used more outpatient consultations (2, 95% CI 2.4 – 2.4) compared to the never married. Education and income had small effects on different aspects of healthcare utilization. For more details, see Additional file 2, Figures S5,S6,S7,S8,S9, and S10.

#### Healthcare costs

The difference in healthcare costs between patients, depending on their type of cancer, varied by NOK 57,893 for secondary healthcare costs (min breast/ max colorectal), by NOK 4,245 for primary healthcare costs (min breast/ max prostate cancer), and by NOK 62,365 for home- and community-based care costs (min lung/ max prostate cancer). However, because age and access to informal care was more strongly associated with patients’ living situation and healthcare utilization, healthcare costs also depended more on peoples age (total cost-range NOK361,363–418,618) and marital status (total cost-range NOK354,100–411,047), than their underlying cause of death (total cost-range NOK341,318- 392,655). There were some differences in the care pathways depending on patients’ income and education. For example, those with high income had higher costs at the secondary healthcare level and lower costs at the home- and community-based care level. However, the total healthcare costs did not differ between groups of high or low income and education. See Additional file 2, Figures S11,S12,S13, and S14.

## Discussion

In the current paper we used national registries to describe the complete care pathways of all cancer decedents in Norway between 2009 and 2013, during their last six months of life. We also examine how the care pathways were influenced by cancer type, individual- and sociodemographic characteristics, and access to informal care.

Findings show that, towards end-of-life, cancer patients spend less time at home, and more time in short-term institutions and in hospitals. Healthcare utilization (and costs) increased towards end-of-life, both at the secondary, primary, and home and community based level. These findings are in line with previous research [3–5, 8]. Accumulated healthcare costs during the 6 last months of life were high, at NOK339,000–430,000 (US$57,626–72,744 or EURO44,067–55,628). Previous estimates on healthcare costs during the last 6 months of life have primarily focused on hospital costs, and estimates should therefore be compared to our estimates for the secondary care level: NOK162,853–267,097 (US$27,685–45,406) (Table 1). These are in the range of previous findings. For example, Chastek et al. (2011) estimated higher hospital costs at end-of-life (US$74,212) for 28,530 commercially insured patients in the United States (US) [3] while Reeve et al. estimated equally high costs (AUD$30,001 or US$32,800), for 4,271 elderly decedents with a cancer history in Australia [8]. Bekleman et al. (2016) estimated hospital costs during the last 6 months of life for those aged > 60 years, at US$21,840 in Canada, US$19,783 in Norway, US$18,500 in the US, US$16,221 in Germany, US$15,699 in Belgium, US$10,936 in the Netherlands, and US$9,342 in England [51]. In these estimates – outpatient visits were excluded. If looking at estimates in countries that differ more from Norway, Zhong et al. (2019) found that the healthcare costs for 894 cancer patients in urban China was US$18,234 while [52] Hung et al., (2018) found that the healthcare costs for 195,228 Taiwanese cancer decedents was US$48,234 [53]. Estimates are likely different between settings for several reasons, among them, variation in the age of the population, the healthcare systems, and the cultures.

Even though the majority of resource use was at the secondary care level (44–66%), a large proportion of resources were also used at the home- and community-based care level (31–52%). Findings indicate that services in the secondary and home- and community-based care setting to a large degree are substitutes; those that had lower healthcare utilization and costs at the secondary care level (e.g., those 90 + compared to the youngest age group, and women compared to men) had higher healthcare utilization and costs at the home- and community based care level, and vice versa; those who had higher costs at the secondary care level (e.g., those who were married compared to the never married) had lower costs in the home- and community based care setting (see Additional file 2, Figures S1,S2,S3,S4,S5,S6,S7,S8,S9,S10,S11,S12,S13, and S14). As a result of our findings, costs in one level of the healthcare sector should never be evaluated alone when estimating the total cost of care at end-of-life or the cost-effectiveness of interventions at end-of-life, since this may lead to biased analyses and wrong conclusions. For example, the total costs in the elderly appear to be substantially lower if only hospital costs are included, than if costs of home- and community based care are also included [8]. Alternatively, an intervention might appear to be cost-effective if it leads to a reduction in hospital costs, but these costs might be outweighed by an increase in costs at the home- and community based care level, leading to the intervention not being cost-effective after all. This pattern of substitution has also been found in populations dying from other causes than cancer [54].

Few other researchers have focused on both healthcare utilization and healthcare costs at cancer patients end-of-life, thus comparisons with other studies are challenging. However, if comparing healthcare utilization in our paper, to findings of people dying from other causes, we observe similar patterns of healthcare utilization during the last months of life. For example, Luta et al. (2020), who performed a retrospective cohort study of individuals aged > 60 years (*n* = 108,510) who died in England between 2010 and 2017, estimated the average no. of hospital admissions, length-of-stay in hospital, and GP-consultations at 0.6, 4.7 and 2.8, respectively [55]. As a comparison, we estimated the use of these healthcare components ranging (min/max) between 0.6/1.0, 3.8/6.7 and 2.3/2.9 (Fig. 2 and Additional file 1, Table S1). This indicates similar healthcare utilization disregarding the cause of death.

We found that type of cancer had an impact on the care pathway of patients; however, patients’ age was more strongly associated with the care pathway than cause of cancer death; higher age was associated with fewer days at home and more days in nursing homes. This pattern was expected, as other factors than cancer, such as frailty, is more prevalent in older age groups.

We also found a strong association between informal care (marital status) and care pathway; those with a partner stayed approximately 30 days more at home—on average—compared to those without a spouse or a partner. However, an important weakness in our paper and in the literature generally, is that we do not measure actual use of informal care (i.e., the number of hours that a partner provides care to his/ her spouse). Another weakness in our paper is that informal care may have been provided by others than a spouse or partner, for example, we found that those who were previously married or with a partner also had a higher number of days at home, compared to the never married. This indicates that informal care from others, in this case most likely children, also influences the care pathways at end-of-life. In younger subpopulations, such as cervical cancer patients, friends or parents probably play an important role as informal caregivers. Future end-of-life research would benefit from an increased focus on actual resource use from informal caregivers, and on the relationship between this informal care and formal care provided at the secondary, primary, and home- and community based care level.

Even though the care pathways differed slightly between individuals with different levels of education and income, we found that the total healthcare utilization (measured in the total costs) did not differ between groups depending on education and income. While this is expected in a society as Norway, where egalitarian values are strong, we might underestimate the influence of sociodemographic factors (such as income) since we only include public healthcare, and not private healthcare. However, Norway has publicly financed healthcare, and coverage is universal and automatic for all residents [56]. Thus, private healthcare spendings are small and not expected to influence our results greatly.

This paper has some further limitations worth mentioning. First, the dataset covers the time-period 2009–2013, which is 8–12 years ago. Structural changes have happened to the healthcare system in Norway that might have influenced the care pathways of patients at their end-of-life. For example, the Coordination Reform implemented in 2012, had as its aim to give more responsibility to the municipalities (primary and home- and community based care providers) to relieve the secondary care providers, and through this, reduce healthcare costs. After the reform, nurses working in the municipalities reported an increase in the number of poorly functioning patients discharged to municipal services [57]. This might have influenced patients at their end-of-life in different ways. The patients at their end-of-life may receive more home-based care following the reform, since the municipalities have been given more responsibility for these types of treatments. Or, since municipalities now have an increased number of other severely ill patients, there might be less time to focus on patients at their end-of-life. A study evaluating the evolution of care at end-of-life from the 1970s to the 1990s by Riley et al., (2010) found that there had been a steady increase in home-based services throughout the last 50 years, thus, this might have continued until now [25]. However, how time (since 2013) has influenced end-of-life care is an empirical question, that we cannot answer in the current paper because of data limitations.

To ensure anonymity, we were not allowed more detailed information regarding time alive since cancer diagnosis than the information in Table 1. Namely, years since diagnosis 0, 1, 2,3, 4, 5, 6–10, 11–20, 20 + . This means that for the patients who lived less than one year, we cannot differentiate between patients who died less or more than 6 months before their cancer diagnosis. For these patients, we still include living situation, healthcare utilization and costs from the last 6 months of their life.

Last, we do not indicate which care pathway that offer high/ low quality to the patient—we simply describe the care that patients receive. In the literature, the number of days that people spend at home has been suggested as an indication of quality [58]. If so, those who have access to informal care seem to receive higher quality care. However, more research is needed on which factors that actually contribute to high-quality care and whether these factors are perceived as quality indicators from both the patients and the informal care-givers perspective.

Despite weaknesses, the current paper has several important strengths; we describe the care pathways for all cancer decedents in Norway between 2009–2013, thus, in a non-selected cohort. We describe where people receive their care (their living situation) and not just their place of death. By estimating healthcare utilization at different levels, our analyses describe the burden of care at end-of-life to the different care providers. We also identified the main components of end-of-life care and describe which components that weigh heavily on the total costs. Since we had access to individual and sociodemographic characteristics, and in addition, could indicate access to informal care, we were able to explain variability (i.e., heterogeneity) in the use of resources. This information can inform future analyses about important characteristics to consider when performing heterogeneity analyses of care at end-of-life.

## Conclusion

In this registry based study, covering all cancer decedents in Norway in the period 2009–2013, we found that cancer patients, on average, spend 123 days at home, 24 days in hospital, 16 days in short-term care and 24 days in long-term care during their last 6 months of life. Healthcare utilization increased towards end-of-life at all care levels, and the secondary (60%) and home- and community-based care providers (37%) bear the largest burden of the total costs. Cancer patient’s care pathways at end-of-life were more associated with age and access to informal care, than underlying type of cancer and sociodemographic characteristics. We also found that care was substituted between the different healthcare levels. Based on this, we suggest that care at end-of-life should never be evaluated at one healthcare level alone, as this might drastically bias results and lead to suboptimal priorities.

## Supplementary Information

**Additional file 1.** 

**Additional file 2.** 

## Data Availability

Restrictions apply to the availability of the data that support the findings of this study, which were used under license for the current study. The data are therefore not available upon request from the authors. They can be made available through formal applications to the Norwegian Directorate of Health, the Norwegian Institute of Public Health, Statistics Norway and the Cancer Registry of Norway.

## Abbreviations

AIC: Akaike information criterion
AME: Average marginal effects
BIC: The Bayesian information criterion
CDR: The Norwegian Causes of Death Registry
CCI: Charlson Comorbidity Index
CRN: The Cancer Registry of Norway
DRG: Diagnosis-related groups
ER: Emergency room
GLM: Generalized linear models
GP: General practitioner
HELFO: The Norwegian Health Economics Administration
IPLOS: The individual-based nursing and care statistics registry called
KOSTRA: The Municipality-State-Reporting
KUHR: The Control and Payment of Health Reimbursement registry
NOK: Norwegian Kroner
NPR: The Norwegian Patient Registry
SSB: Statistics Norway

## Footnotes

1 Cervical cancer was included for two purposes, despite low incidence rates. First, because this patient population is distinctly different from many other cancer populations as it is often diagnosed in young females. Thus, we believe that including these as a separate group will increase our understanding of the contribution of gender and age when we contrast findings between the different groups of cancer decedents. Second, cervical cancer was also included because it has large focus among policy makers since it is a cancer many countries aim at preventing or detecting at an early stage through vaccination and screening.

2 ER is here defined as out-of-hour consultations that patients use if they need acute consultation with a medical doctor outside of office hours. It is not care at an emergency room in the hospital. In the ER, as defined here, conditions are often more severe than conditions treated at the GP within office hours.
