# Supplementary material for: Care pathways at end-of-life for cancer decedents: registry based analyses of the living situation, healthcare utilization and costs for all cancer decedents in Norway in 2009-2013 during their last 6 months of life
Source: BMC Health Serv Res. 2022 Oct 1;22:1221. doi: 10.1186/s12913-022-08526-w (PMC9526273; doi:10.1186/s12913-022-08526-w)
Supplement: Supplementary file 1 — Additional file 1. [file 12913_2022_8526_MOESM1_ESM.pdf]

Additional file

Care pathways at end-of-life for cancer decedents.

Registry based analyses of the living situation, healthcare utilization and costs for all cancer decedents in Norway in 2009-2013 during their last 6 months of life.

Gudrun Bjørnelv[1,2], Terje Hagen[1], Leena Forma[3,4] and Eline Aas[1,5]

[1] Department of Health Management and Health Economics, Institute of Health and Society, University of Oslo, Oslo, Norway

[2] Department of Public Health and Nursing, Norwegian University of Science and Technology, Trondheim, Norway

[3] Faculty of Social Sciences, Tampere University, Tampere, Finland

[4] Laurea University of Applied Sciences, Vantaa, Finland

[5] Division for Health Services, Institute of Public Health, Oslo, Norway

Corresponding author: Gudrun Maria Waaler Bjørnelv (gudrun.m.w.bjornelv@ntnu.no). ORCID identifier: 0000-0003-4997-5426

Additional file 1

Table S1: Average number of treatments/ days that patients receive *per month*, in the time period 6-4 months, 3-2 months and 1 month before death. Corresponding to Figures 1 and 2 in the manuscript.

	All (n=52,926)		Lunge (n=8,701)		Colorectal (n=6,468)		Prostate (n=4,658)		Breast (n=3,123)		Cervix (n=353)		Other (n=29,623)	
	#	SD	#	SD	#	SD	#	SD	#	SD	#	SD	#	SD
**Secondary care**														
(6-4 months before death)														
Inpatient consultations	0.42	(0.84)	0.37	(0.71)	0.36	(0.72)	0.39	(0.87)	0.36	(0.68)	0.58	(0.73)	0.45	(0.91)
Outpatient consultations	1.23	(1.87)	1.24	(1.84)	1.40	(1.94)	0.98	(1.44)	1.60	(2.03)	1.51	(2.61)	1.19	(1.88)
Days in hospital	2.20	(4.14)	1.98	(3.59)	1.91	(3.64)	1.88	(3.50)	1.79	(3.56)	3.29	(5.21)	2.41	(4.49)
(3-2 months before death)														
Inpatient consultations	0.74	(1.02)	0.73	(0.90)	0.66	(0.92)	0.64	(1.00)	0.62	(0.87)	1.01	(1.02)	0.79	(1.09)
Outpatient consultations	1.40	(2.14)	1.54	(2.22)	1.38	(2.05)	0.99	(1.64)	1.68	(2.30)	1.63	(2.59)	1.39	(2.17)
Days in hospital	4.93	(6.71)	4.91	(6.26)	4.53	(6.45)	3.79	(5.53)	4.02	(6.02)	6.68	(7.91)	5.28	(7.07)
(1 month before death)														
Inpatient consultations	1.22	(1.33)	1.30	(1.27)	1.10	(1.31)	1.01	(1.28)	1.11	(1.21)	1.18	(1.19)	1.27	(1.36)
Outpatient consultations	1.20	(2.39)	1.45	(2.68)	1.15	(2.28)	0.83	(1.94)	1.31	(2.43)	1.37	(2.92)	1.19	(2.38)
Days in hospital	7.45	(8.20)	8.27	(8.27)	6.88	(7.95)	5.51	(7.25)	6.73	(7.94)	7.59	(8.57)	7.70	(8.34)
**Primary care**														
(6-4 months before death)														
GP consultations	1.76	(1.79)	1.81	(1.74)	1.59	(1.66)	2.16	(2.04)	1.49	(1.69)	1.89	(2.06)	1.74	(1.78)
ER visits	0.14	(0.34)	0.14	(0.35)	0.12	(0.28)	0.21	(0.42)	0.10	(0.31)	0.14	(0.30)	0.13	(0.33)
(3-2 months before death)														
GP consultations	2.31	(2.43)	2.47	(2.36)	2.15	(2.32)	2.67	(2.69)	1.84	(2.16)	2.28	(2.54)	2.28	(2.45)

ER visits	0.25	(0.53)	0.27	(0.56)	0.22	(0.48)	0.34	(0.64)	0.18	(0.41)	0.25	(0.48)	0.24	(0.52)
(1 month before death)														
GP consultations	2.70	(3.95)	2.83	(3.77)	2.71	(4.01)	2.92	(4.17)	2.27	(3.79)	2.46	(4.18)	2.68	(3.96)
ER visits	0.75	(1.26)	0.78	(1.33)	0.73	(1.22)	0.91	(1.34)	0.64	(1.09)	0.63	(1.12)	0.73	(1.25)
**Home and community based care**														
(6-4 months before death)														
Days in longtime institution	3.50	(9.51)	1.94	(7.25)	4.04	(10.13)	5.22	(11.19)	5.28	(11.34)	3.15	(9.00)	3.39	(9.38)
Days in shorttime institution	1.30	(4.76)	1.13	(4.56)	1.27	(4.68)	1.87	(5.54)	1.33	(4.68)	1.39	(4.97)	1.26	(4.70)
Hours practical assistance	1.52	(12.13)	1.17	(8.67)	1.66	(13.33)	1.17	(6.26)	2.38	(18.22)	1.15	(6.21)	1.55	(12.65)
Hours nursing care	6.60	(21.91)	4.40	(14.67)	7.06	(22.26)	8.61	(24.13)	8.75	(25.67)	9.21	(25.96)	6.58	(22.68)
(3-2 months before death)														
Days in longtime institution	4.19	(10.25)	2.43	(7.93)	4.66	(10.76)	6.47	(12.16)	6.08	(12.01)	3.68	(9.80)	4.06	(10.10)
Days in shorttime institution	2.75	(6.85)	2.60	(6.69)	2.67	(6.81)	3.72	(7.76)	2.60	(6.73)	3.04	(7.48)	2.67	(6.74)
Hours practical assistance	1.61	(12.33)	1.33	(9.61)	1.75	(13.97)	1.24	(7.20)	2.24	(13.07)	1.49	(7.42)	1.66	(13.23)
Hours nursing care	8.97	(25.46)	6.60	(19.16)	9.48	(25.67)	10.82	(26.69)	10.83	(28.27)	12.47	(30.38)	9.02	(26.39)
(1 month before death)														
Days in longtime institution	4.91	(10.96)	3.01	(8.74)	5.30	(11.33)	7.67	(13.04)	6.86	(12.55)	3.90	(10.06)	4.75	(10.82)
Days in shorttime institution	6.81	(10.84)	6.74	(10.68)	6.95	(10.96)	7.84	(11.38)	6.12	(10.49)	7.31	(11.57)	6.71	(10.79)
Hours practical assistance	1.64	(12.45)	1.33	(9.51)	1.74	(13.53)	1.27	(7.56)	2.32	(14.03)	1.63	(8.56)	1.69	(13.41)
Hours nursing care	12.06	(31.62)	9.69	(25.55)	12.97	(32.88)	13.19	(31.91)	13.02	(32.23)	15.11	(35.38)	12.25	(32.74)
**Days at home**														
(6-4 months before death)	23.79	(10.66)	25.71	(8.90)	23.57	(11.00)	21.83	(11.91)	22.37	(11.85)	23.05	(10.50)	23.74	(10.63)
(3-2 months before death)	19.46	(11.78)	21.36	(10.72)	19.46	(11.99)	17.33	(12.53)	18.57	(12.49)	18.25	(11.47)	19.34	(11.75)
(1 month before death)	12.50	(11.30)	13.60	(11.06)	12.47	(11.41)	10.69	(11.55)	11.85	(11.39)	12.78	(11.51)	12.53	(11.26)
**Healthcare costs**														

Secondary healthcare	226,394	(198,205)	222,328	(168,739)	234,442	(186,270)	162,830	(144,943)	198,428	(171,834)	267,141	(205,876)	238,289	(215,487)
Primary care	12,285	(13,143)	12,748	(12,693)	11,429	(12,376)	14,520	(14,306)	9,458	(11,267)	11,862	(14,923)	12,287	(13,335)
Home and communitybased care	143,036	(200,124)	103,899	(165,627)	153,018	(206,344)	195,138	(212,381)	179,504	(223,781)	148,905	(203,537)	140,239	(200,733)
Total healthcare costs	381,714	(244,071)	338,976	(215,152)	398,889	(234,607)	372,487	(217,924)	387,390	(224,038)	427,908	(252,102)	390,814	(258,037)
