# Supplementary material for: Care pathways at end-of-life for cancer decedents: registry based analyses of the living situation, healthcare utilization and costs for all cancer decedents in Norway in 2009-2013 during their last 6 months of life
Source: BMC Health Serv Res. 2022 Oct 1;22:1221. doi: 10.1186/s12913-022-08526-w (PMC9526273; doi:10.1186/s12913-022-08526-w)
Supplement: Supplementary file 2 — Additional file 2. [file 12913_2022_8526_MOESM2_ESM.pdf]

# Additional file

**Care pathways at end-of-life for cancer decedents.**

Registry based analyses of the living situation, healthcare utilization and costs for all cancer decedents in Norway in 2009-2013 during their last 6 months of life.

Gudrun Bjørnelv[1,2], Terje Hagen[1], Leena Forma[3,4] and Eline Aas[1,5]

[1] Department of Health Management and Health Economics, Institute of Health and Society, University of Oslo, Oslo, Norway

[2] Department of Public Health and Nursing, Norwegian University of Science and Technology, Trondheim, Norway

[3] Faculty of Social Sciences, Tampere University, Tampere, Finland

[4] Laurea University of Applied Sciences, Vantaa, Finland

[5] Division for Health Services, Institute of Public Health, Oslo, Norway

Corresponding author: Gudrun Maria Waaler Bjørnelv (gudrun.m.w.bjornelv@ntnu.no). ORCID identifier: 0000-0003-4997-5426

Additional file 2 – Figures illustrating results from the regression analyses.

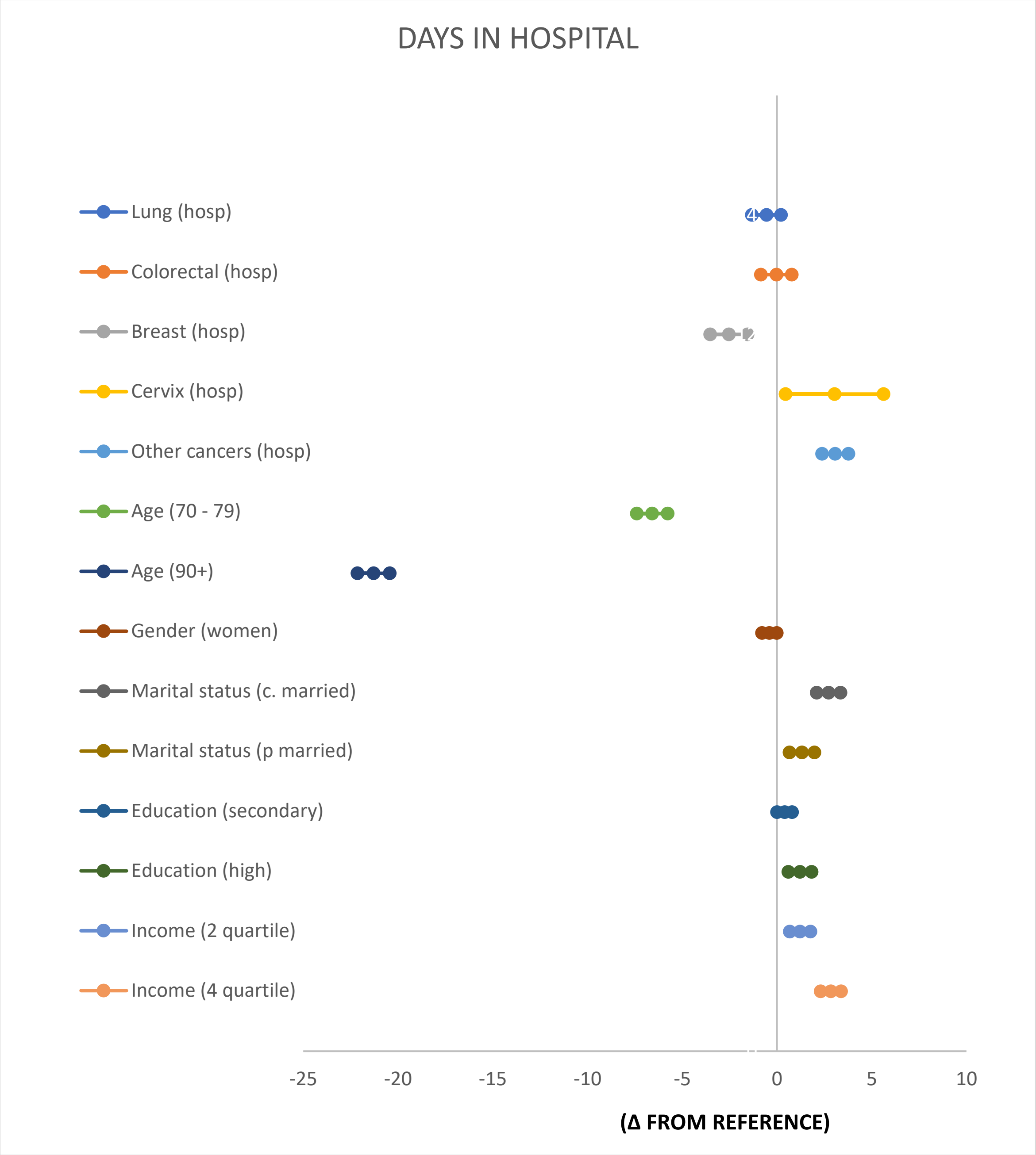


***Figure S1: strength of the association, and confidence interval, when regressing the number of days in hospital on the underlying cause of death, age, gender, access to informal care (marital status), education and income of individuals. The variables charlson index, time of death (year) and time since cancer diagnosis are included in the regression, but not shown in the Figure. Numbers show the difference, and confidence interval of the difference, from the reference category*.**

** c. married = currently married, p.married = previously married.*

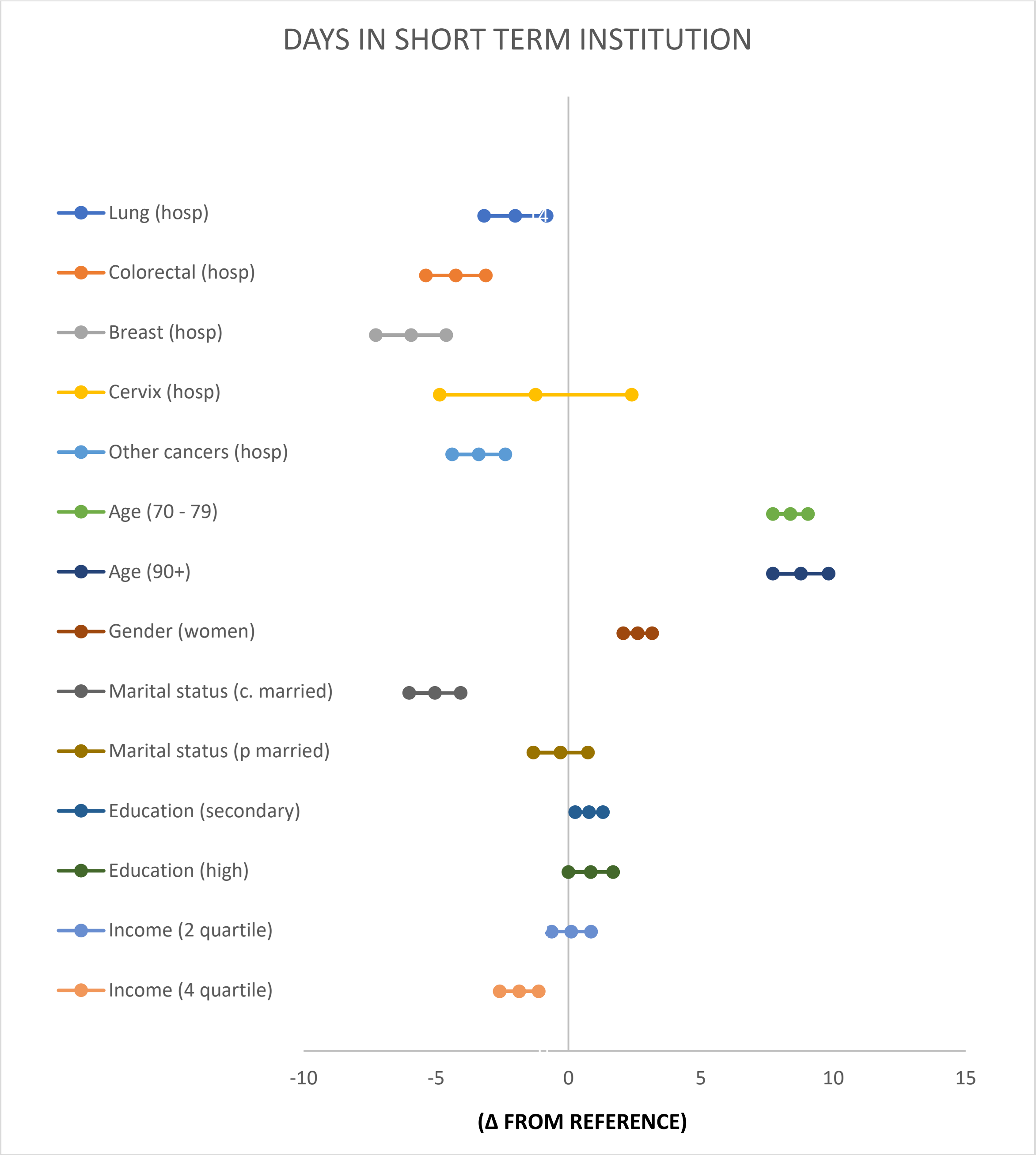


***Figure S2: strength of the association, and confidence interval, when regressing the number of days in short-term institution on the underlying cause of death, age, gender, access to informal care (marital status), education and income of individuals. The variables charlson index, time of death (year) and time since cancer diagnosis are included in the regression, but not shown in the Figure. Numbers show the difference, and confidence interval of the difference, from the reference category*.**

** c. married = currently married, p.married = previously married.*

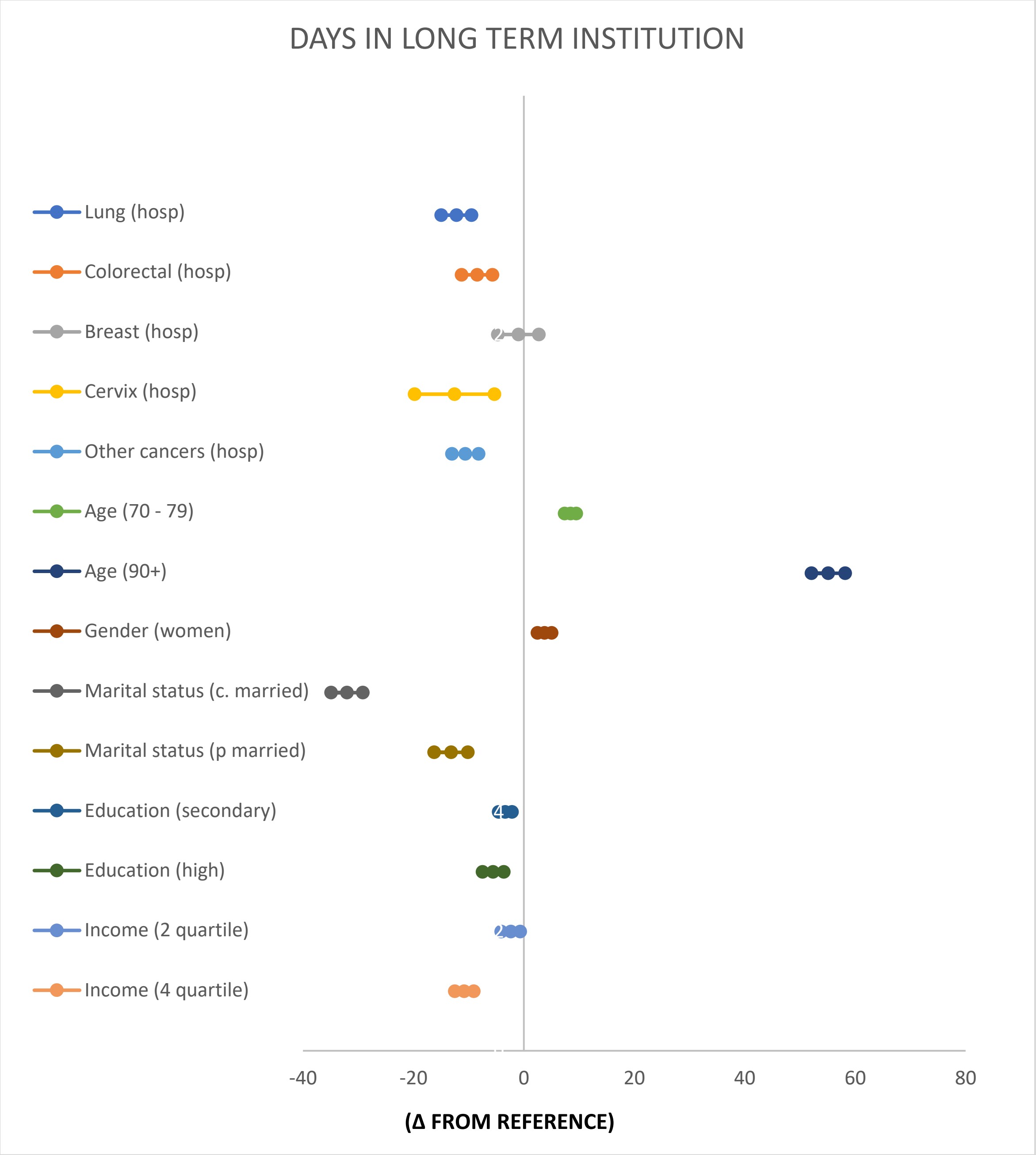


***Figure S3: strength of the association, and confidence interval, when regressing the number of days in long-term institution on the underlying cause of death, age, gender, access to informal care (marital status), education and income of individuals. The variables charlson index, time of death (year) and time since cancer diagnosis are included in the regression, but not shown in the Figure. Numbers show the difference, and confidence interval of the difference, from the reference category*.**

** c. married = currently married, p.married = previously married.*

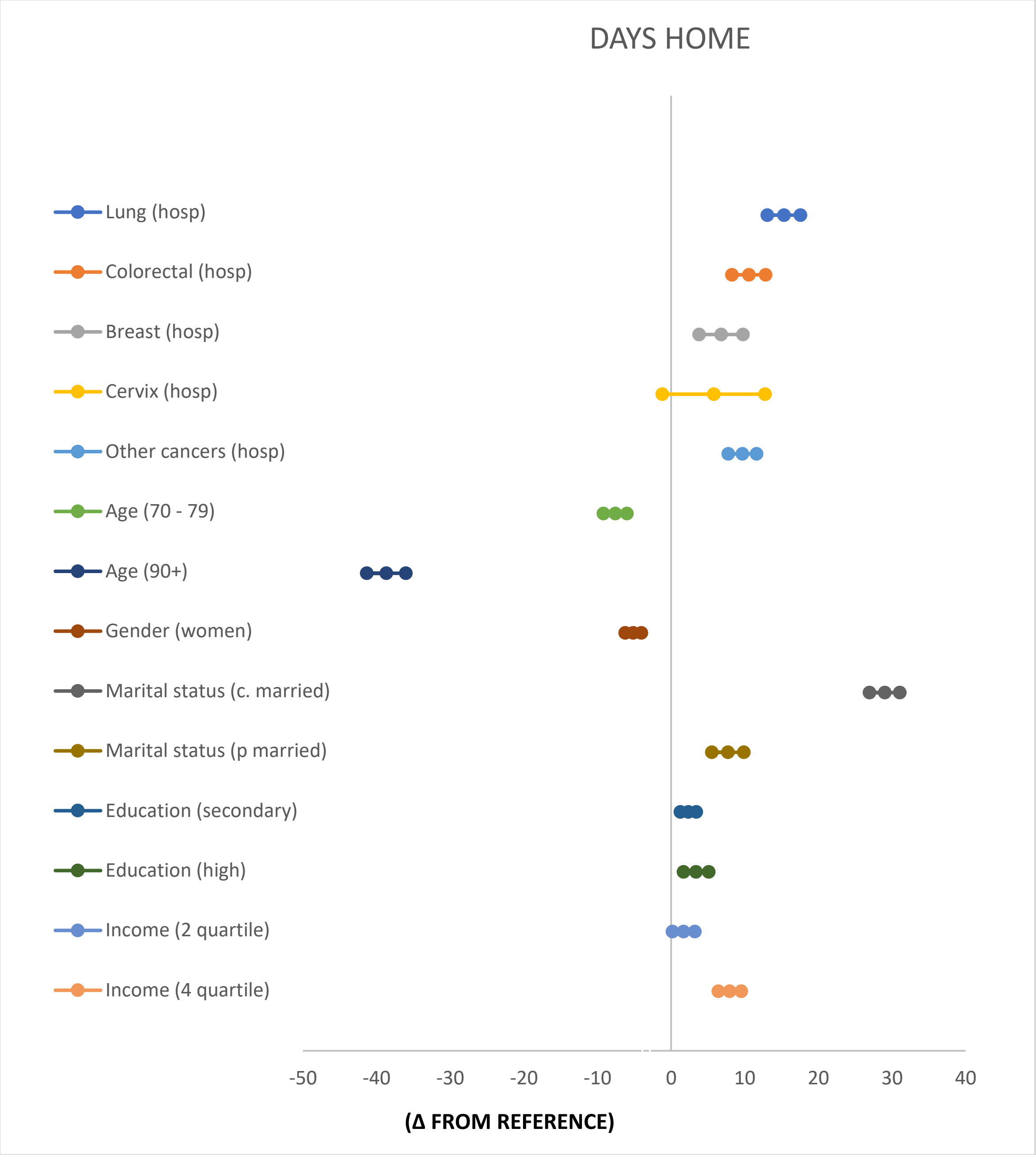


***Figure S4: strength of the association, and confidence interval, when regressing the number of days at home on the underlying cause of death, age, gender, access to informal care (marital status), education and income of individuals. The variables charlson index, time of death (year) and time since cancer diagnosis are included in the regression, but not shown in the Figure. Numbers show the difference, and confidence interval of the difference, from the reference category***.

** c. married = currently married, p.married = previously married.*

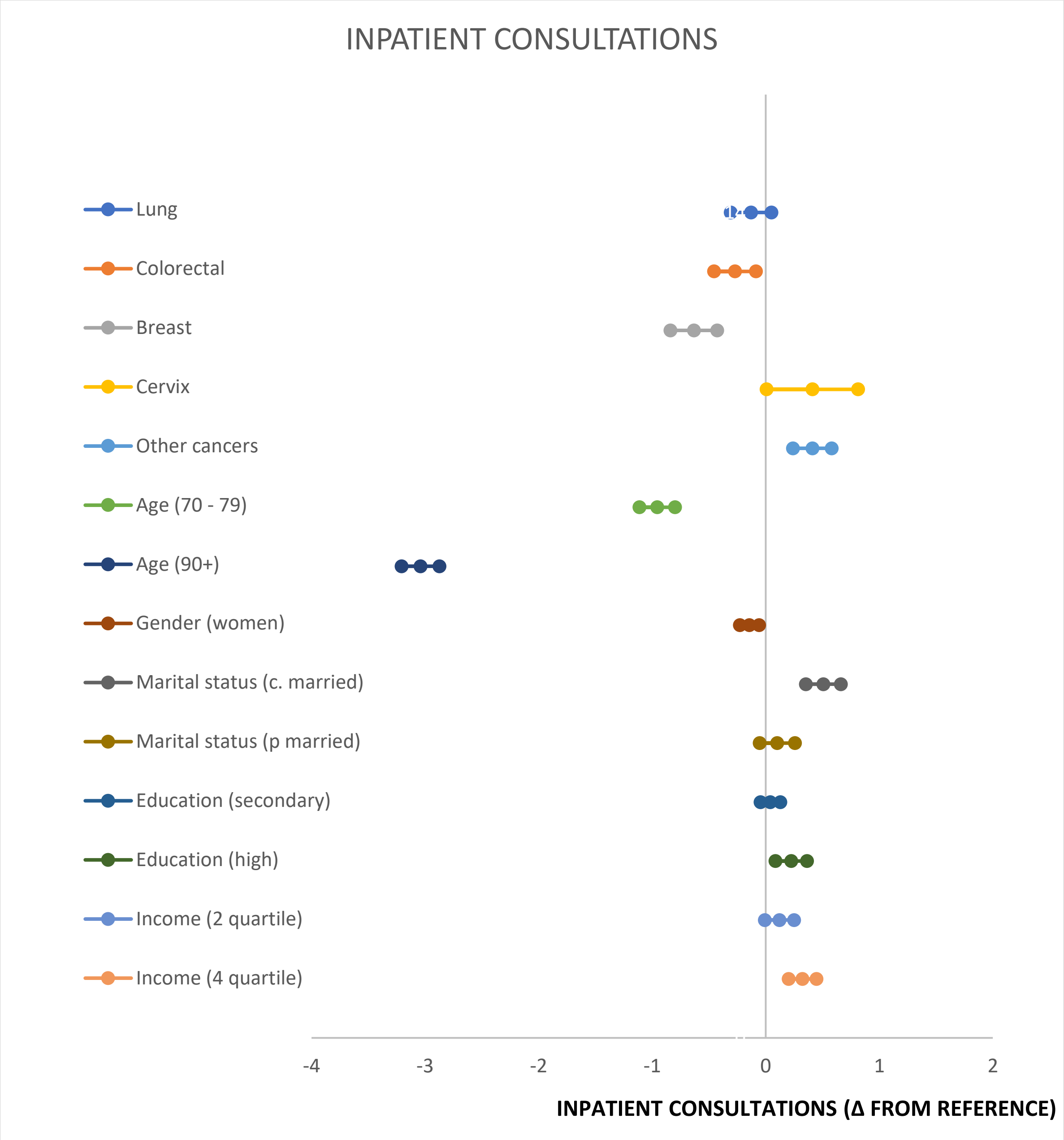


***Figure S5: strength of the association, and confidence interval, when regressing the number of inpatient consultations on the underlying cause of death, age, gender, access to informal care (marital status), education and income of individuals. The variables charlson index, time of death (year) and time since cancer diagnosis are included in the regression, but not shown in the Figure. Numbers show the difference, and confidence interval of the difference, from the reference category*.**

** c. married = currently married, p.married = previously married.*

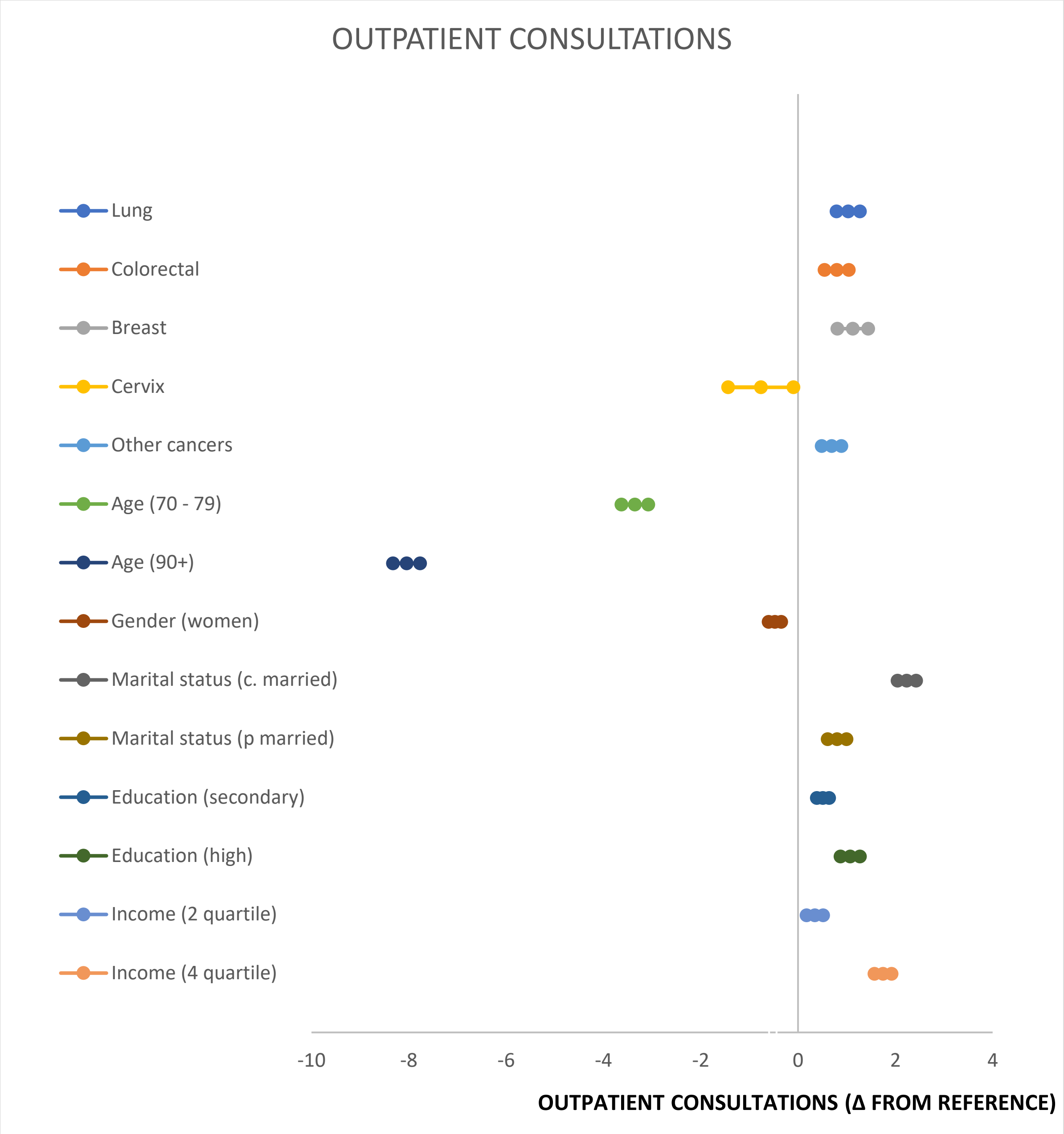


***Figure S6: strength of the association, and confidence interval, when regressing the number of outpatient consultations on the underlying cause of death, age, gender, access to informal care (marital status), education and income of individuals. The variables charlson index, time of death (year) and time since cancer diagnosis are included in the regression, but not shown in the Figure. Numbers show the difference, and confidence interval of the difference, from the reference category***.

** c. married = currently married, p.married = previously married.*

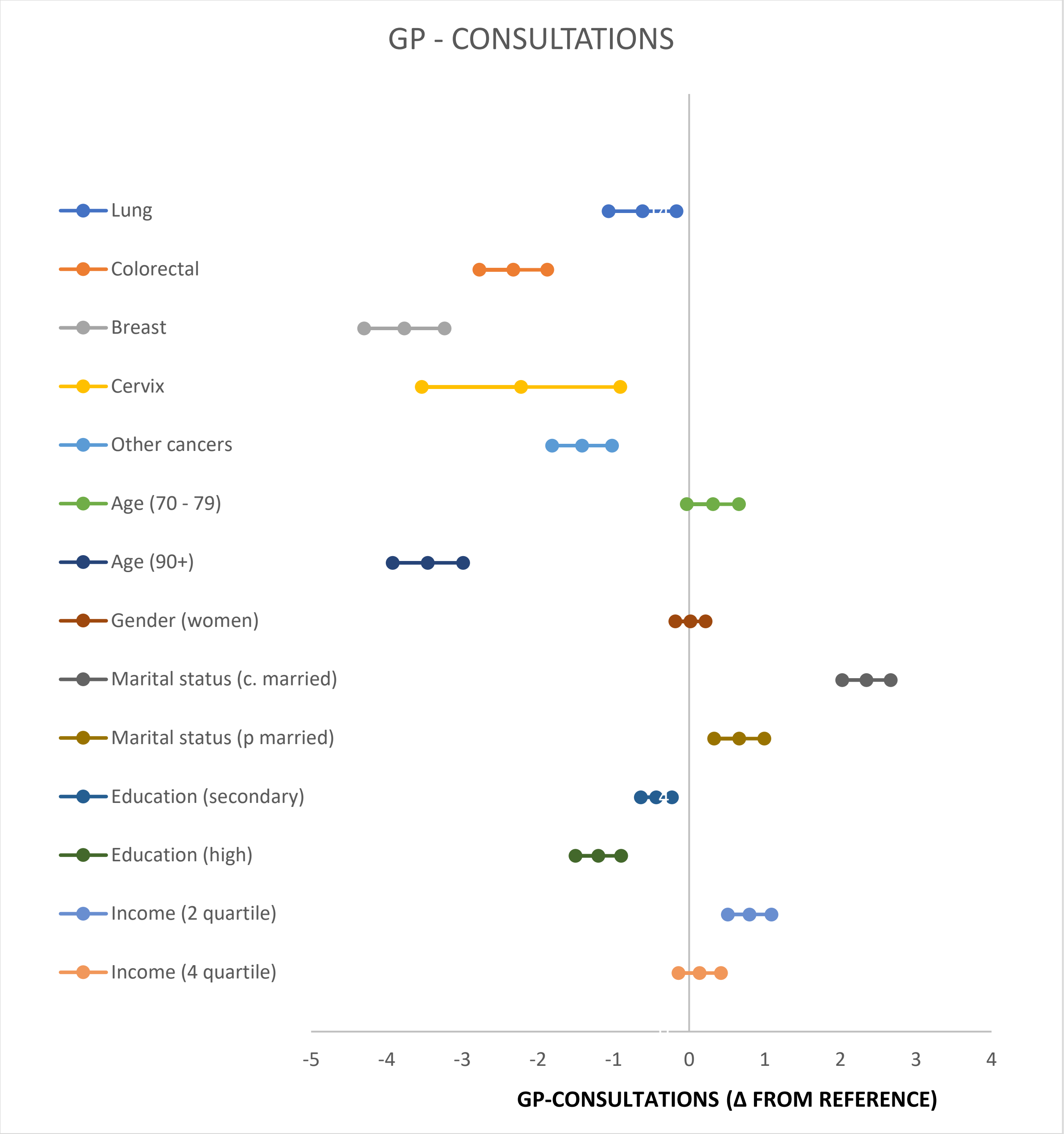


***Figure S7: strength of the association, and confidence interval, when regressing the number of GP-consultations on the underlying cause of death, age, gender, access to informal care (marital status), education and income of individuals. The variables charlson index, time of death (year) and time since cancer diagnosis are included in the regression, but not shown in the Figure. Numbers show the difference, and confidence interval of the difference, from the reference category*.**

** c. married = currently married, p.married = previously married.*

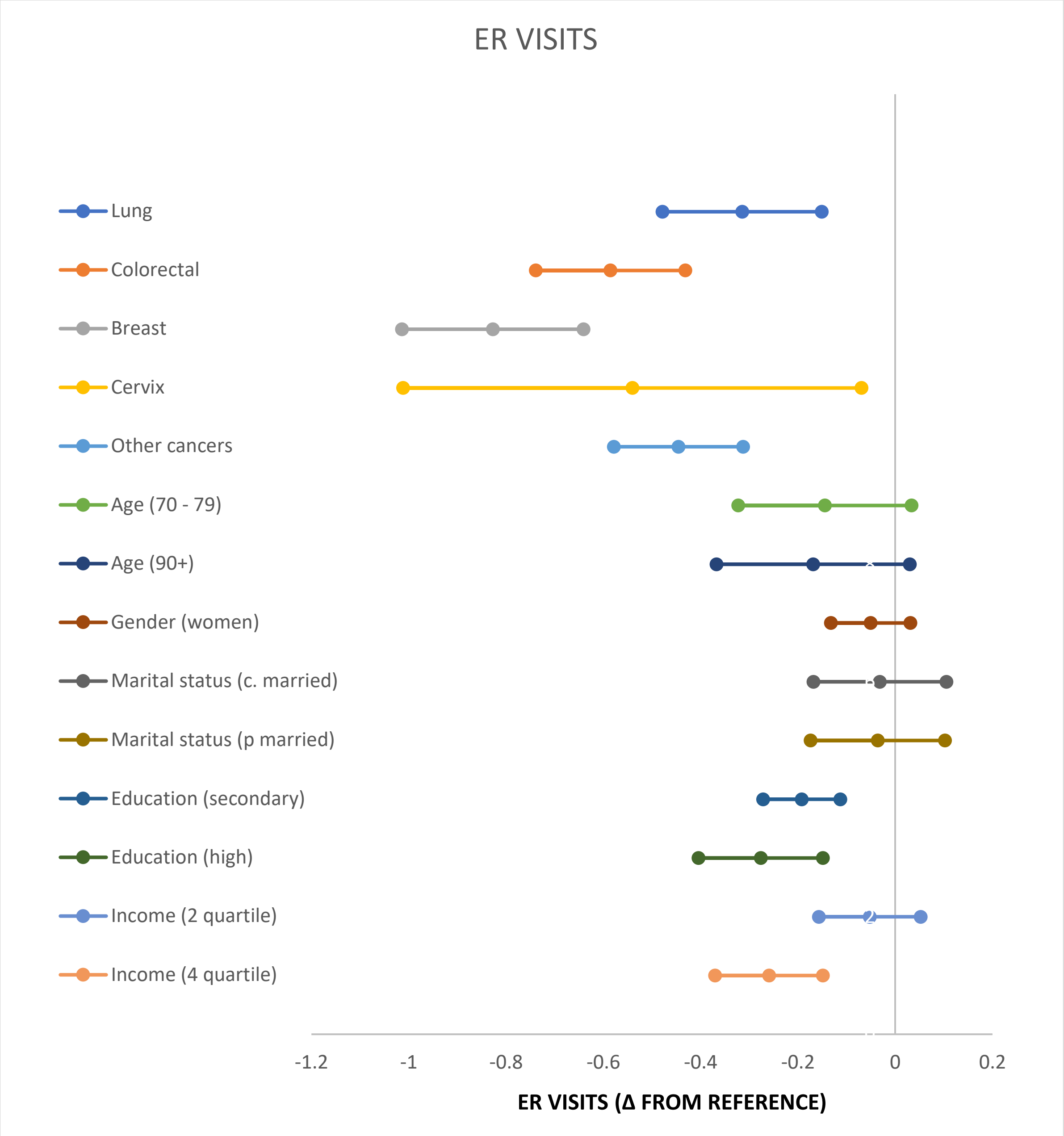


***Figure S8: strength of the association, and confidence interval, when regressing the number of ER-visits on the underlying cause of death, age, gender, access to informal care (marital status), education and income of individuals. The variables charlson index, time of death (year) and time since cancer diagnosis are included in the regression, but not shown in the Figure. Numbers show the difference, and confidence interval of the difference, from the reference category*.**

** c. married = currently married, p.married = previously married.*

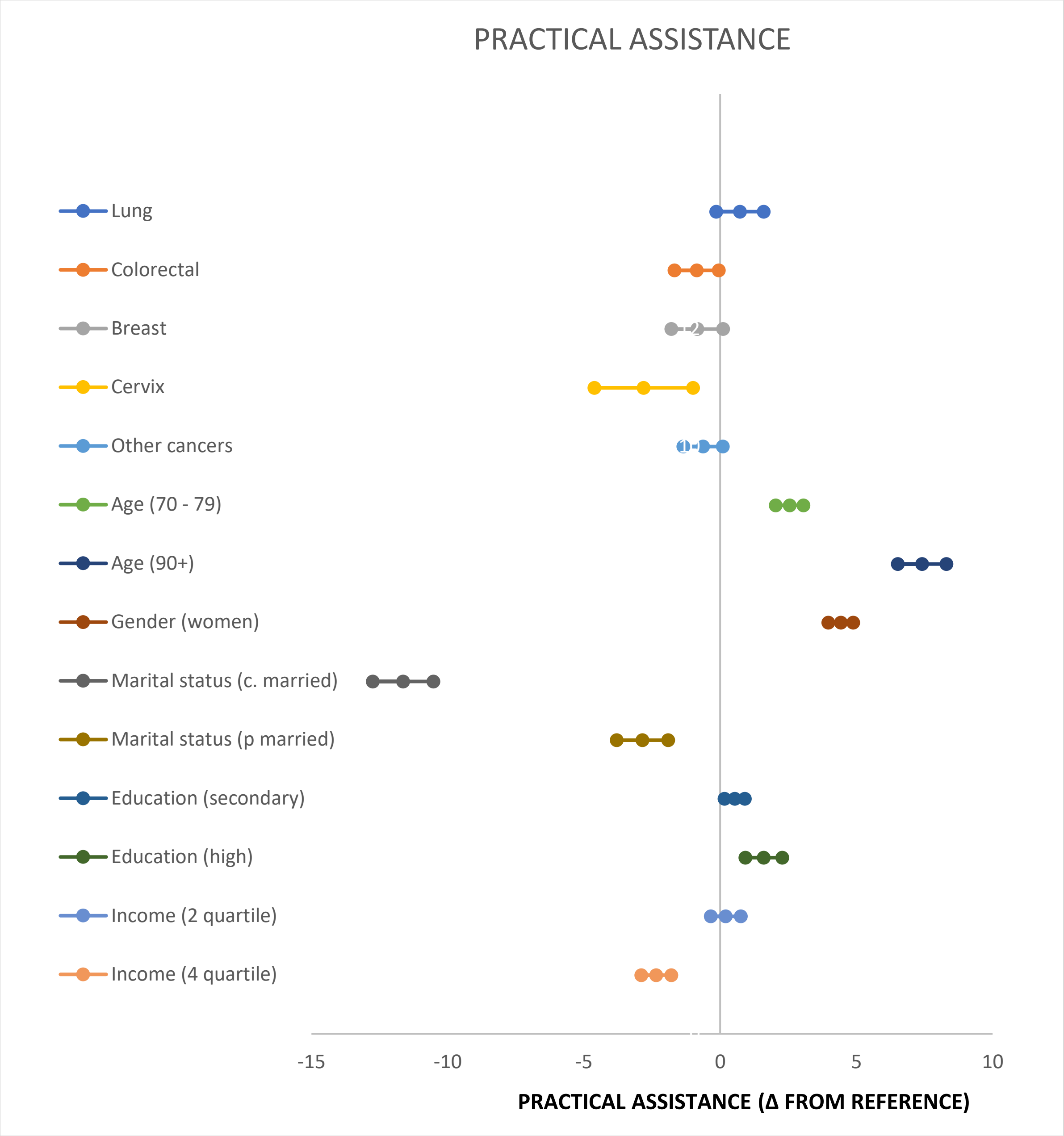


***Figure S9: strength of the association, and confidence interval, when regressing the number of hours of practical assistance on the underlying cause of death, age, gender, access to informal care (marital status), education and income of individuals. The variables charlson index, time of death (year) and time since cancer diagnosis are included in the regression, but not shown in the Figure. Numbers show the difference, and confidence interval of the difference, from the reference category*.**

** c. married = currently married, p.married = previously married.*

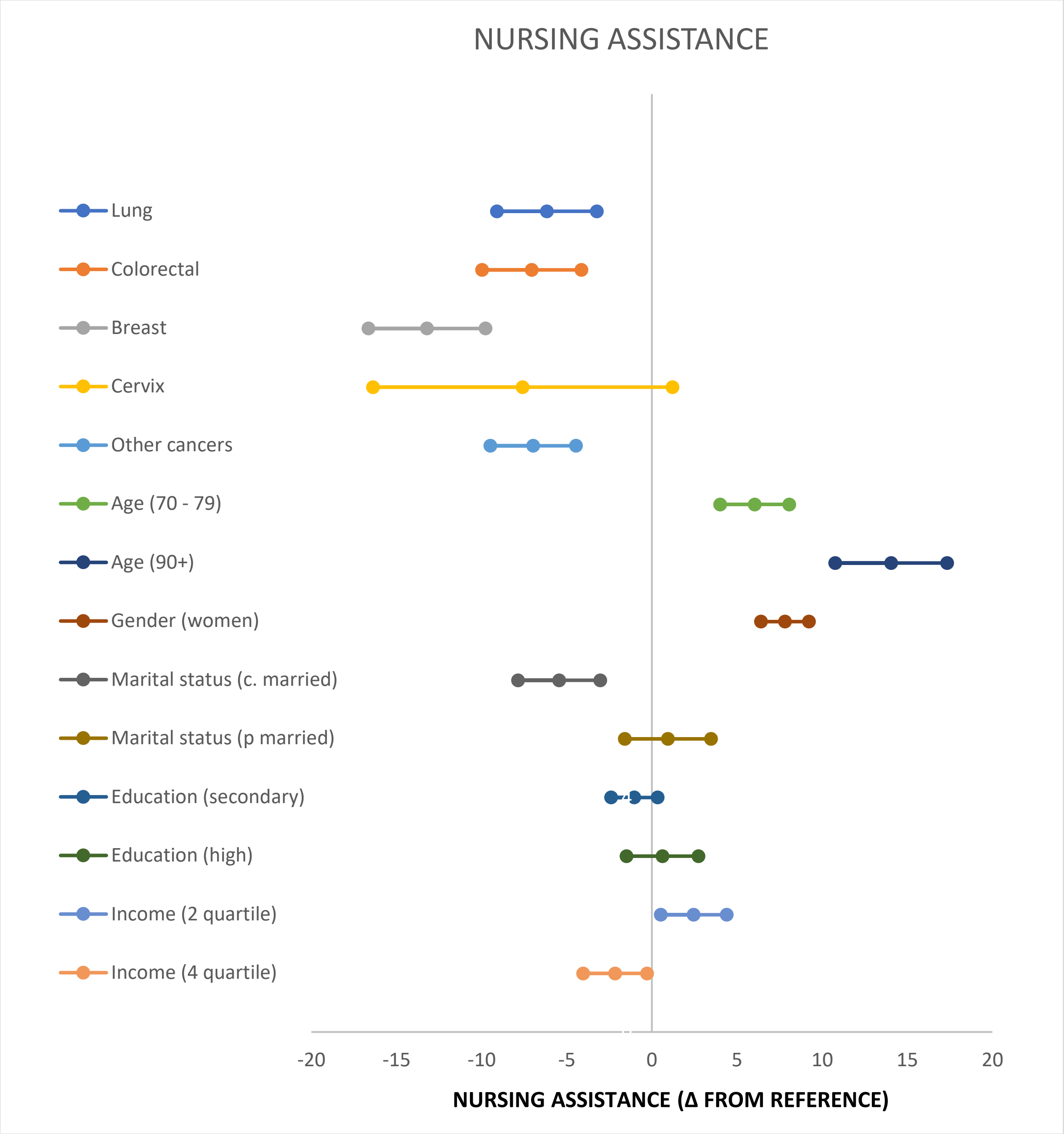


***Figure S10: strength of the association, and confidence interval, when regressing the number of hours of nursing assistance on the underlying cause of death, age, gender, access to informal care (marital status), education and income of individuals. The variables charlson index, time of death (year) and time since cancer diagnosis are included in the regression, but not shown in the Figure. Numbers show the difference, and confidence interval of the difference, from the reference category***.

** c. married = currently married, p.married = previously married.*

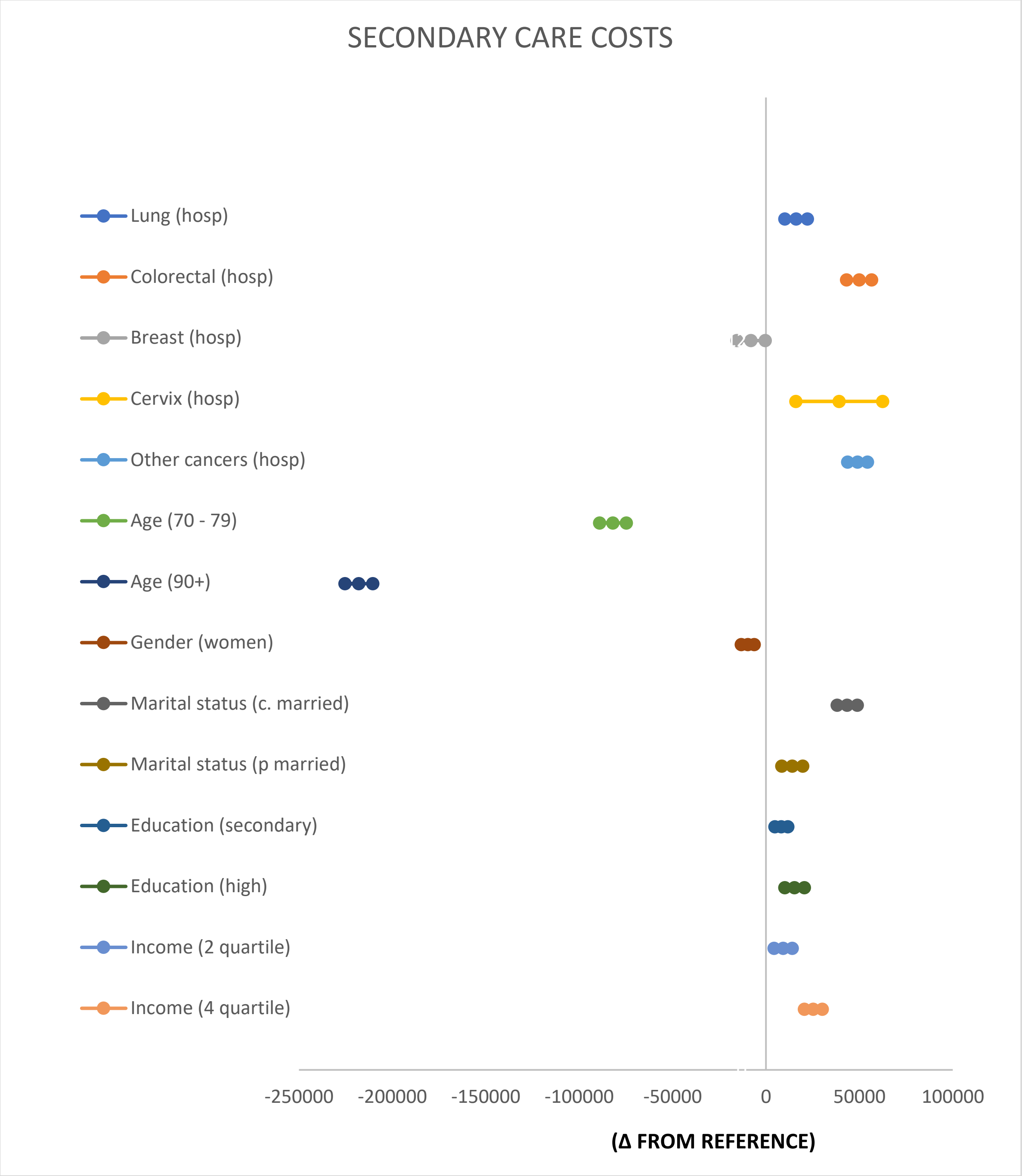


***Figure S11: strength of the association, and confidence interval, when regressing the secondary care costs (during the last 6 months of life) on the underlying cause of death, age, gender, access to informal care (marital status), education and income of individuals. The variables charlson index, time of death (year) and time since cancer diagnosis are included in the regression, but not shown in the Figure. Numbers show the difference, and confidence interval of the difference, from the reference category.***

** c. married = currently married, p.married = previously married.*

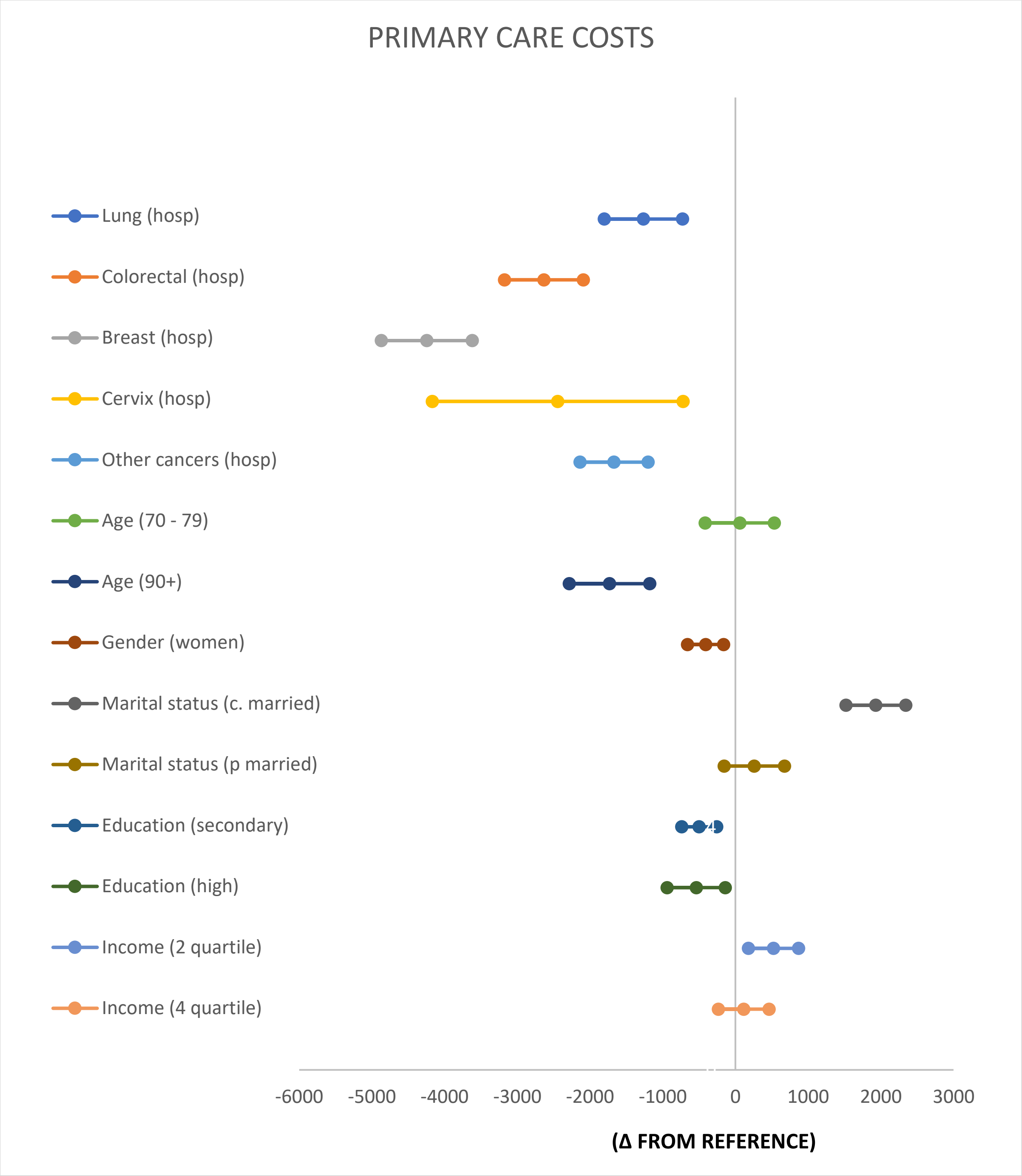


***Figure S12: strength of the association, and confidence interval, when regressing the primary care costs (during the last 6 months of life) on the underlying cause of death, age, gender, access to informal care (marital status), education and income of individuals. The variables charlson index, time of death (year) and time since cancer diagnosis are included in the regression, but not shown in the Figure. Numbers show the difference, and confidence interval of the difference, from the reference category*.**

** c. married = currently married, p.married = previously married.*

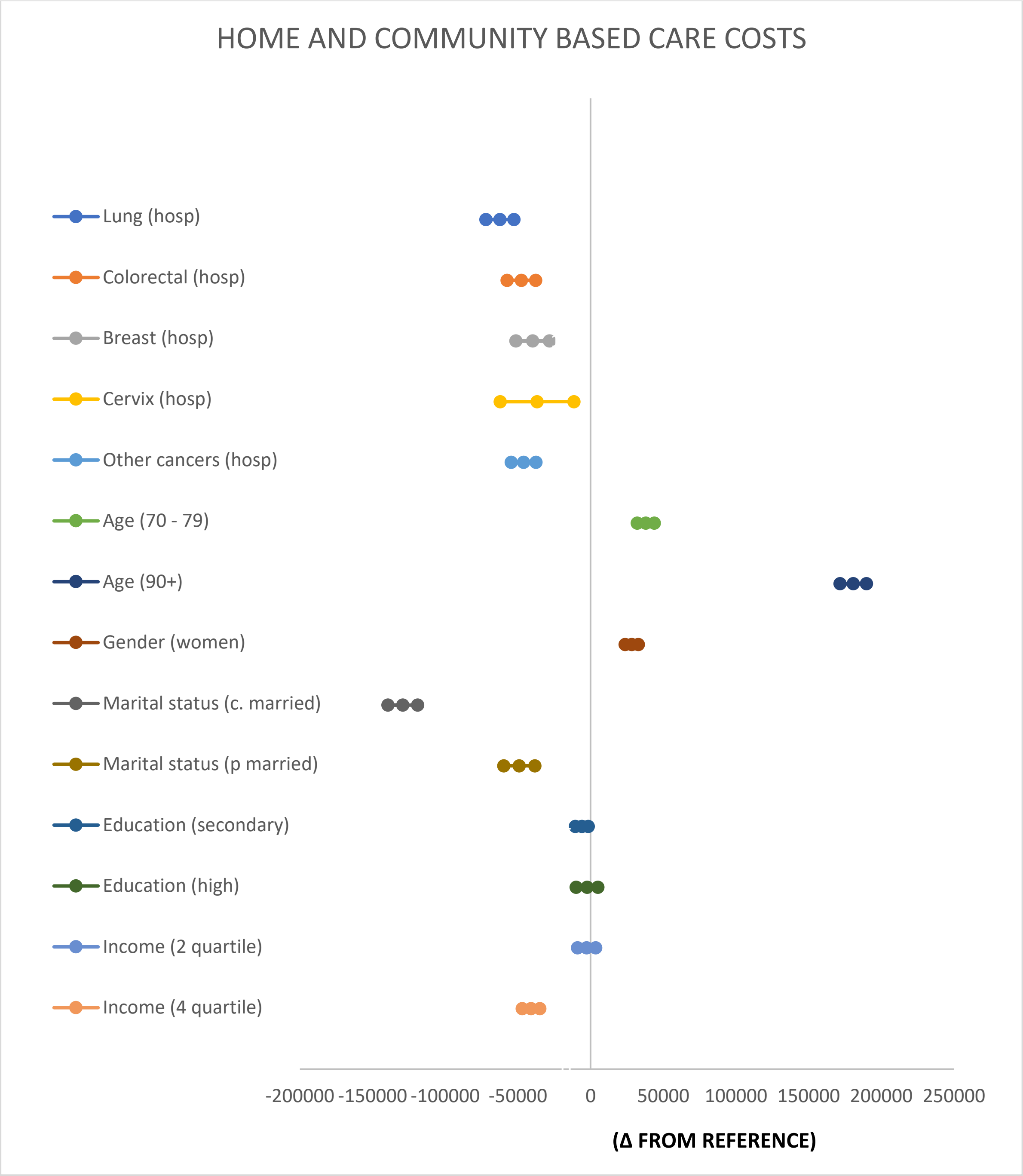


***Figure S13: strength of the association, and confidence interval, when regressing the home- and comminuty based care costs (during the last 6 months of life) on the underlying cause of death, age, gender, access to informal care (marital status), education and income of individuals. The variables charlson index, time of death (year) and time since cancer diagnosis are included in the regression, but not shown in the Figure. Numbers show the difference, and confidence interval of the difference, from the reference category*.**

** c. married = currently married, p.married = previously married.*

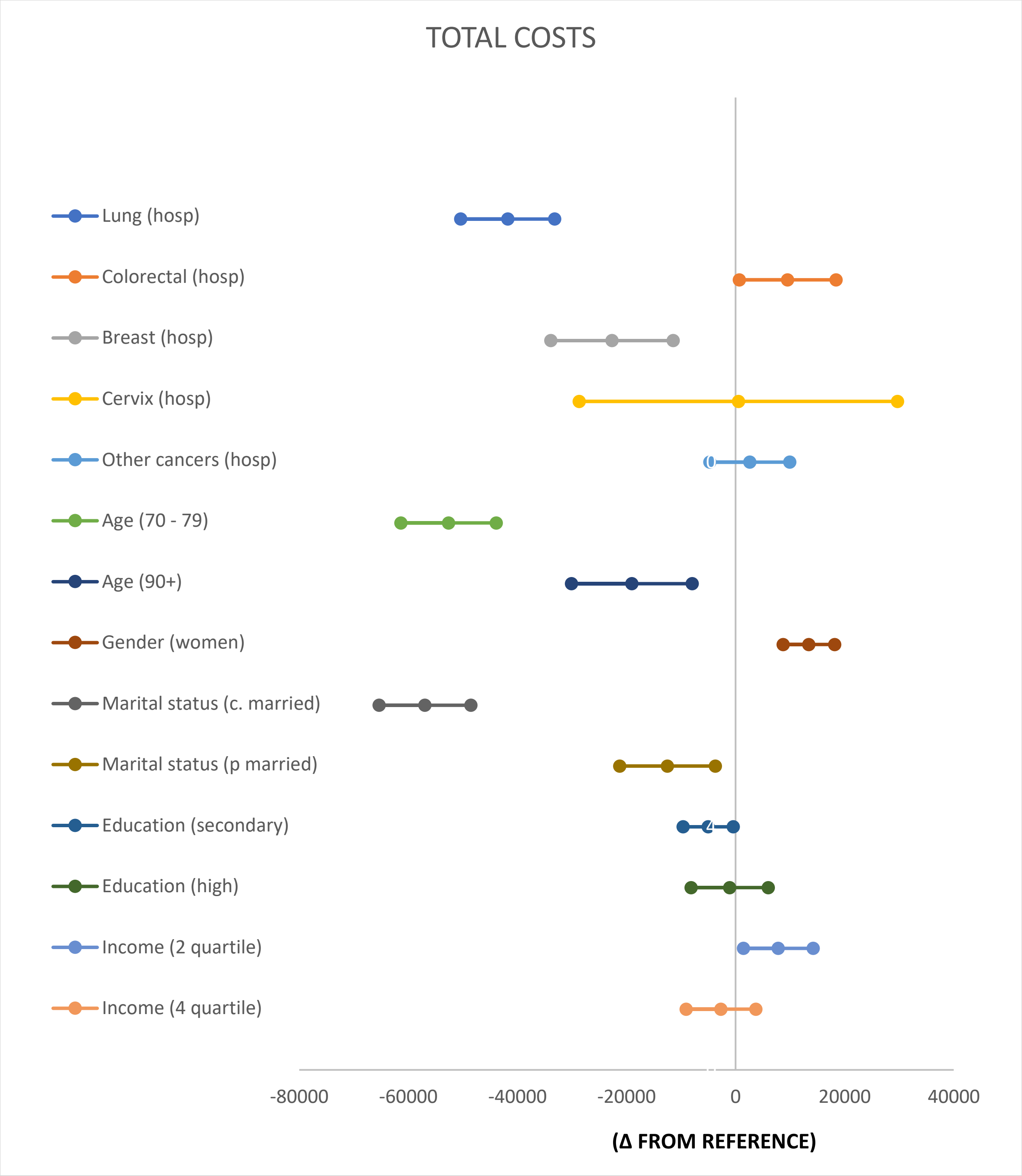


***Figure S14: strength of the association, and confidence interval, when regressing the total costs of care (during the last 6 months of life) on the underlying cause of death, age, gender, access to informal care (marital status), education and income of individuals. The variables charlson index, time of death (year) and time since cancer diagnosis are included in the regression, but not shown in the Figure. Numbers show the difference, and confidence interval of the difference, from the reference category*.**

** c. married = currently married, p.married = previously married.*
